# Microwave-assisted multicomponent reactions in heterocyclic chemistry and mechanistic aspects

**DOI:** 10.3762/bjoc.17.71

**Published:** 2021-04-19

**Authors:** Shivani Gulati, Stephy Elza John, Nagula Shankaraiah

**Affiliations:** 1 Department of Medicinal Chemistry, National Institute of Pharmaceutical Education and Research (NIPER), Hyderabad 500 037, India

**Keywords:** cycloaddition, Knoevenagel condensation, Michael addition, microwave, multicomponent reactions

## Abstract

Microwave-assisted (MWA) multicomponent reactions (MCRs) have successfully emerged as one of the useful tools in the synthesis of biologically relevant heterocycles. These reactions are strategically employed for the generation of a variety of heterocycles along with multiple point diversifications. Over the last few decades classical MCRs such as Ugi, Biginelli, etc. have witnessed enhanced yield and efficiency with microwave assistance. The highlights of MWA-MCRs are high yields, reduced reaction time, selectivity, atom economy and simpler purification techniques, such an approach can accelerate the drug discovery process. The present review focuses on the recent advances in MWA-MCRs and their mechanistic insights over the past decade and shed light on its advantage over the conventional approach.

## Introduction

Recently, organic chemists are focussed to develop environment friendly sustainable technologies and procedures using atom-efficient reactions from suitable starting materials to meet the demands of present as well as future generations [1–3]. The need arises as the traditional method of synthesis has become unsustainable both from an environmental and economic perspective due to increased amounts of waste generation, toxic solvents, and no real-time control of pollution generated, etc. [4]. Therefore, in this connection, the multicomponent reaction (MCR) is one such approach where three or more reactants combine to form a single product retaining the majority of the atoms of the starting materials. The ability of forming multiple bonds in one-pot via a multicomponent reaction provides a novel and sustainable method in drug discovery [4]. In the recent years, these reactions have emerged as a promising strategy following green chemistry principles such as reduction in waste generation, step-economy, minimum use of solvents, along with atom and bond-forming economy. In addition, MCRs are eco-friendly with simple purification procedures, faster reactions favoring chemo- and regioselectivity in some cases [5–7]. The multicomponent strategy has provided an easy access to the synthesis of complex bioactive molecules with multiple point diversity incorporating up to eight components in one-pot [8]. The pharmaceutical industry has witnessed a considerable surge in drug synthesis via multicomponent strategies [9–11], including the synthesis of atorvastatin, a potent HMG-CoA reductase inhibitor. Dömling and co-workers efficiently demonstrated an Ugi-based MCR approach towards the synthesis of atorvastatin in four steps ruling out the lengthy seven step protocol, and paving a rapid entry to the drug discovery market [12].

Alternatively, microwave-assisted (MWA) organic synthesis marked its presence on the scientific map in 1986 with two reports of organic syntheses in the kitchen microwave [13–14]. This paved a new direction in synthetic chemistry wherein technology was incorporated to achieve the desired results adroitly. The golden decade of MWA organic synthesis (2000–10), witnessed microwaves with optic fibre or IR pyrometers for temperature detection along with specific glass reaction vessels that can withstand pressure and temperature in the reaction generated especially by low boiling solvents. Microwave-assisted heating reduces reaction time from hours to minutes and seconds. It provides efficient and uniform heating proving to be a rapid method over the conventional ones. Reactants are directly heated when microwave heating is employed while with the conventional methods, the reaction vessel is first heated and the heat is transferred through convection to the participating reactants [15–19]. Over the years, microwave reactors have undergone considerable changes making it adaptable at various levels of organic synthesis. Primarily, there are three types of microwave reactors namely monomode microwave reactors, multimode microwave reactors for parallel synthesis and multimode microwave reactors for single-batch scale-up. The reactors vary in capacity and the distribution mode of the electromagnetic wave in the reactor vessel. The introduction of the Si–C (silicon–carbon) vials enables high temperature resistance and selective heating of the heterogeneous catalyst [20–23].

Even though the MWA technology is advantageous, a major challenge posed is the scale-up at the industry level where protocol efficiency at kilogram scale is mandatory. With rapid heat generation, and litres of solvents often safety seems to be compromised imposing restrictions on using microwaves at a scale-up level. However, the batch process and continuous flow process seem to provide an entry into scale-up standards with safety. The microwave reactors serving the purpose of batch and parallel approach are designed in various capacity and modes to achieve the uphill task easily [19].

The contemporary organic chemistry procedure involving microwave heating has paved way for the molecular diversity that helps in reducing the time required for the drug discovery process. As multicomponent reactions and microwave reactions hold their respective advantage over other synthetic protocols, merging strategy with technology proves to be an asset in organic synthesis. In view of the same, chemists worldwide have experimented with the combination which has proved to be highly efficient and sustainable. Over the past decade, researchers have focused on developing greener synthetic strategies for the construction of various pharmacophores which can prove to be vital in a drug discovery process [24–28]. These efforts have not gone unnoticed and have been shaped into reviews in 2010, 2011 by Jiang and Orru respectively [29–30], focusing on the synthetic aspect of five, six, seven and dicyclic structures. Later in 2013, Gupta et al. compiled reports of microwave-assisted cross-coupling, MCR with few cycloaddition reactions [31]. During the course of writing this review, we realized the very presence of two reviews by K. Kamanna and G. Anilkumar highlighting the progressive efforts in MWA-MCRs [32–33]. Recently, Dolzhenko centered a book chapter around named MCR assisted by microwave irradiations [34]. Similarly, in the recent past our research group [35] focussed on unveiling microwave reactions for non-fused single nitrogen-containing heterocycles. A mechanistic understanding of a reaction progression promotes better conceptualization of strategies effectively. This review aims at bridging the hiatus of the previous reviews with mechanistic insights into the MWA-MCRs employed for the synthesis of organic and medicinally significant molecules. The review has been classified on the basis of the pharmacophores constructed by adopting the MWA-MCRs strategy.

## Review

### 1 Acridine

**Figure 1:**
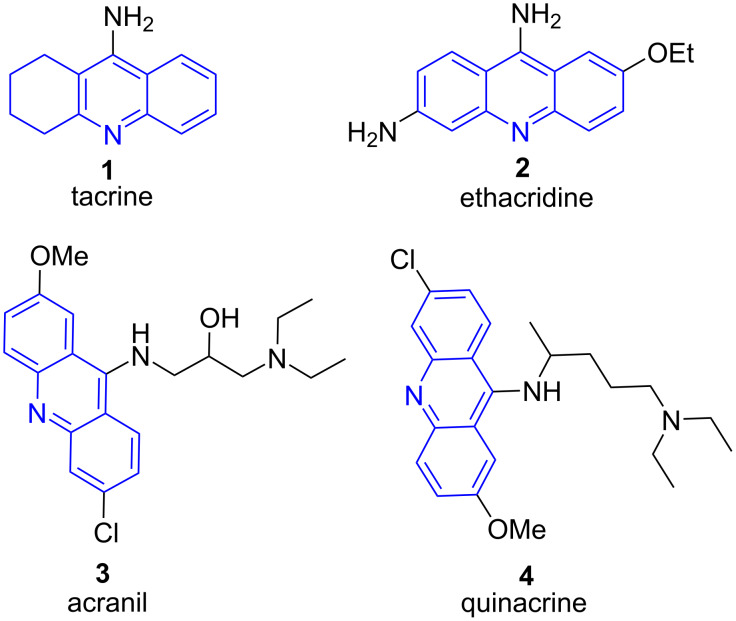
Marketed drugs with acridine moiety.

Acridine is a polycyclic heteroarene with structural basis as anthracene in which one of the central carbon atoms is replaced by a nitrogen atom. Tacrine (**1**) is an acridine derivative used in the treatment of Alzheimer’s disease. A plethora of acridine derivatives have been synthesized and clinically proved with various biological activities such as ethacridine (antibacterial drug; **2**), acranil (antiviral drug; **3**) and quinacrine (antimalarial agent; **4**, Figure 1) [36–37].

**Scheme 1:**
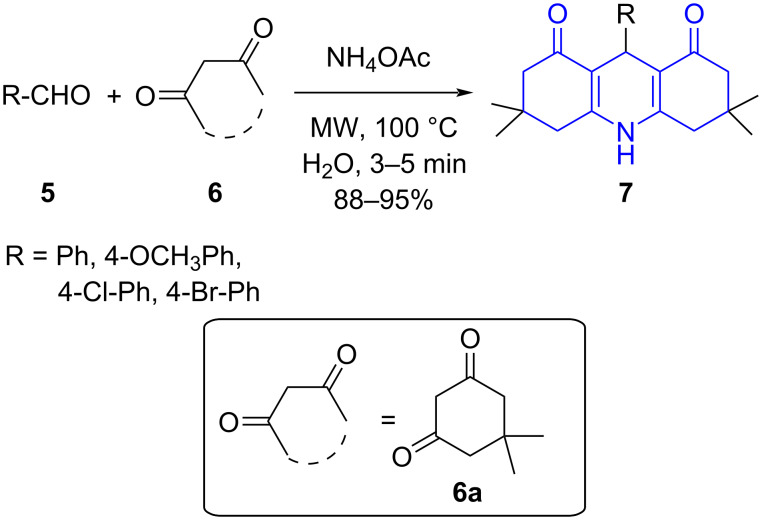
Synthesis of 4-arylacridinediones.

**Scheme 2:**
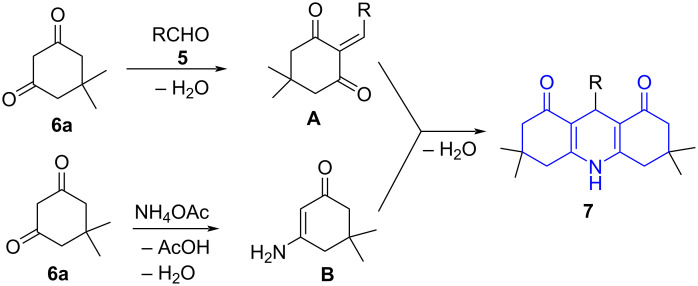
Proposed mechanism for acridinedione synthesis.

The relevance of acridine in drug discovery galvanized Singh and co-workers [38] to develop a water-promoted three-component reaction involving aldehydes **5**, cyclic 1,3-diketone **6** and ammonium acetate powered by microwave irradiation resulting in 4-arylacridinediones **7** in moderate to good yields under catalyst-free conditions (Scheme 1). A rationale of mechanism proposed the transformation via a Knoevenagel condensation between aldehyde and a molecule of **6** affording **A**. The concurrent condensation of ammonium acetate with another molecule of **6** led to the formation of an enaminone **B**. Later, the successive Michael addition of **A** and enaminone **B** followed by an intramolecular cycloaddition with concomitant dehydration delivered the final product **7** (Scheme 2).

**Scheme 3:**
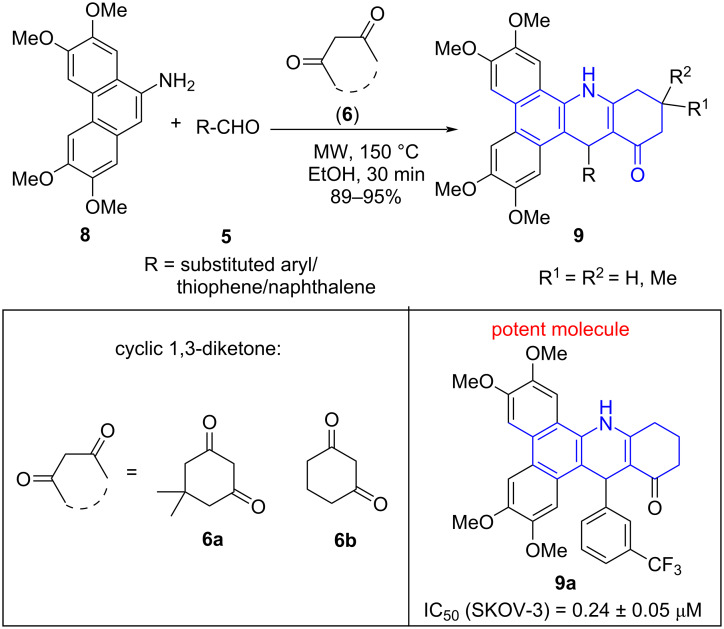
Synthesis of tetrahydrodibenzoacridinones.

In 2018, our research group [39] contemplated and developed an expeditious process for the synthesis of phenanthrene-fused tetrahydrodibenzoacridinones **9** using phenanthren-9-amine **8**, aldehydes **5**, and cyclic 1,3-diketones **6** as structural units in ethanol under microwave irradiation to result in the targeted products in excellent yields. A conventional heating used for the same protocol delivered the desired products in longer reaction time (3 h) with lower yields (60%) as compared to microwave (20 min with 91% yield). The library of molecules synthesized was found to be active against SKOV-3 cancer cells with **9a** emerging as a promising molecule with IC_50_ = 0.24 ± 0.05 μM (Scheme 3). The protocol surfaces the efficiency of MWA-MCR in the construction of fused polycycles with functional diversity for the generation of a library of pharmacologically active molecules.

**Scheme 4:**
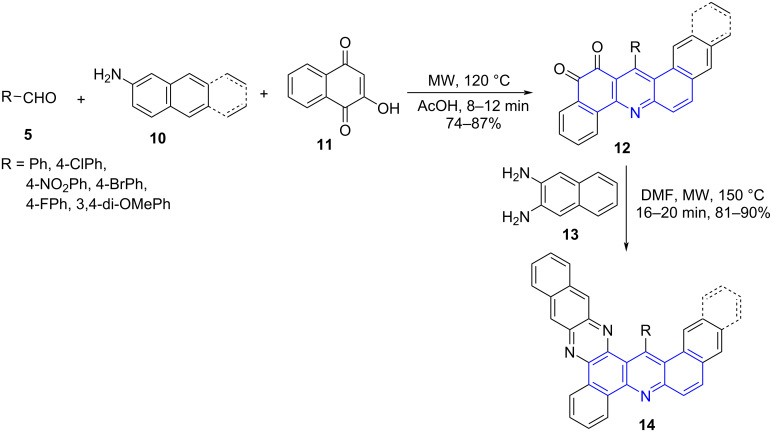
Synthesis of naphthoacridines.

The construction of fused annulated rings are seldomly reported often achieved by a sequential addition approach [40]. Contributing to the same and exploring the MC-MWA reactions Jiang and co-workers [41] designed a microwave facilitated regioselective four-component domino reaction employing naphthyl- or anthracenylamine **10**, aldehydes **5** and 2-hydroxy-1,4-naphthoquinone (**11**) in acetic acid for the construction of dibenzo[*a*,*h*]acridine-12,13(7*H*,14*H*)-dione **12**. The subsequent reaction of benzo[*h*]naphtho[2,3-*a*]acridines **12** with 2,3-diaminonaphthalene (**13**) using DMF as solvent afforded benzophenazine-fused benzacridine **14**. The protocol provided an easy access to extended annular molecules (Scheme 4).

**Scheme 5:**
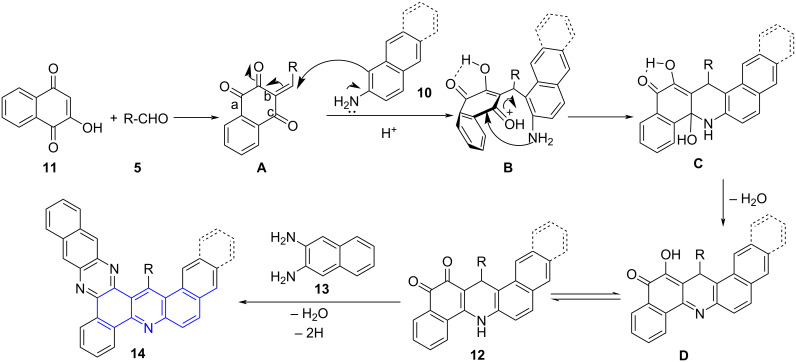
Plausible mechanism for naphthoacridines.

A plausible mechanism as shown in Scheme 5 suggested the involvement of an elementary formation of Knoevenagel adduct **A** from the reaction between the aldehyde and **11**. This adduct undergoes an intermolecular Michael addition to naphthylamine resulting in the formation of **B**. A subsequent intramolecular nucleophilic cyclization leads **C** followed by dehydration forms **D** and finally **12**. The synthesized naphthoacridines **12** with 2,3-diaminonaphthalene produces **14** via dehydration and dehydrogenation.

### 2 Azepines

**Figure 2:**
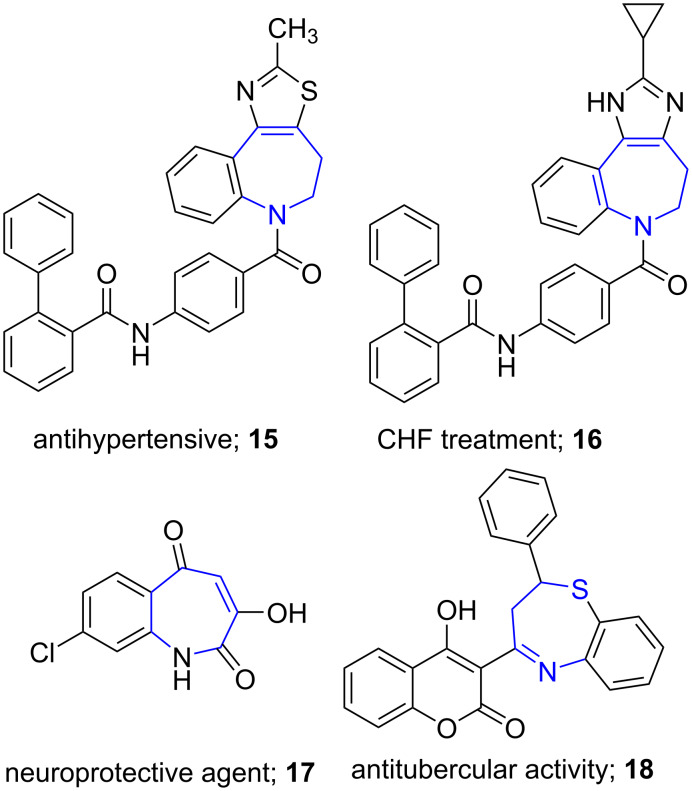
Benzoazepines based potent molecules.

Azepines are represented by unsaturated seven atom heterocyles with nitrogen replacing a carbon atom. The benzene-fused azepines known as benzoazepines have marked their importance in the treatment of various disorders, such as in hypertension (**15**) and in congestive cardiac failure (**16**). They are also known for their use as neuroprotective (**17**) and antitubercular agents (**18**, Figure 2) [42–44].

**Scheme 6:**
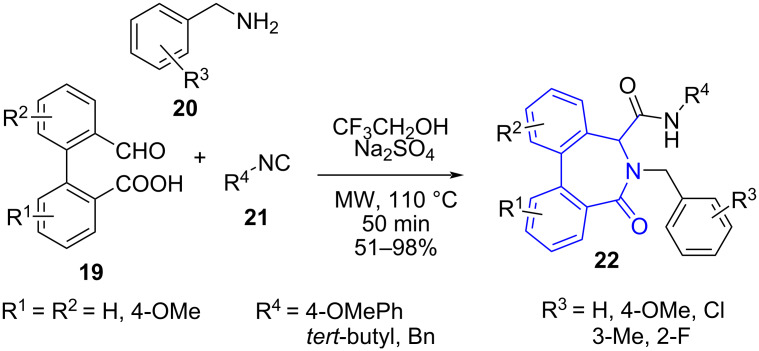
Synthesis of azepinone.

In 2011, Van der Eycken and co-workers [45] tailored a microwave-assisted multicomponent reaction for fast and efficient generation of diastereoselective dibenzo[*c*,*e*]azepinones. The protocol utilized substituted 2'-formylbiphenyl-2-carboxylic acid **19**, benzylamines **20**, and isocyanides **21** in TFE and Na_2_SO_4_ as drying agent for the construction of azepinone **22** and exemplified a modified Ugi reaction (four-component reaction). The aldehyde and acid component of the Ugi reaction was functionalized on the same biaryl ring employing a Suzuki–Miyaura coupling. The authors advocated the use of microwave as it consistently increased the yield from 49% to 82% along with drastic reduction in side product formation and reaction time from 24 h to 50 min when compared to the conventional method. The method proved to be efficient even with chiral amino acids resulting in separable diastereomeric mixtures. The synthesized molecules manifested potent anti-proliferative activity against tumor cell lines leading to the discovery of new lead compounds (Scheme 6).

**Scheme 7:**
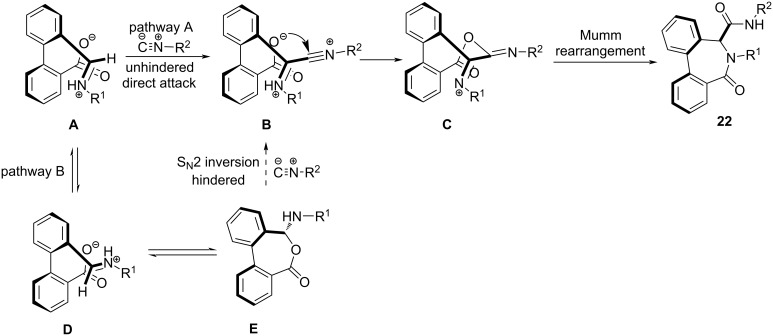
Proposed mechanism for azepinone formation.

A tentative mechanism in Scheme 7 depicts the formation of iminium ion **A** from the reaction between **19** and **20** after the intramolecular protonation by carboxylic acid. The **A** conformer stabilized by electrostatic interaction between carboxylate and iminium moieties undergoes a nucleophilic attack by isocyanide to generate nitrilium ion **B**. The intramolecular acylation of **B** forms **C** followed by Mumm rearrangement results in the formation of the desired products **22**. The intermediate **D** may exist in equilibrium with *N,O*-acetyl intermediate **E**, this may lead to the formation of a very hindered intermediate **C** via S_N_2 inversion of **B** and thus favors the involvement of pathway A rather than pathway B.

**Scheme 8:**
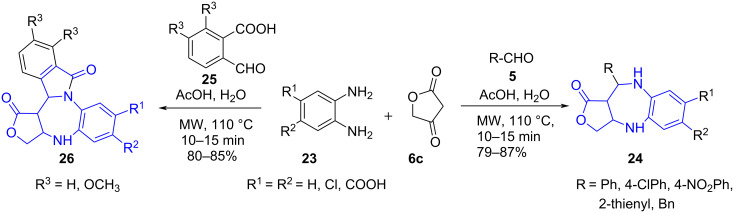
Synthesis of benzoazulenen-1-one derivatives.

Simultaneously, Li and co-workers [46] reported a three-component reaction for the synthesis of benzo[*f*]azulen-1-ones **24** using substituted phenylenediamine **23**, aldehydes **5** and cyclic 1,3-diketone such as tetronic acid **6c** under microwave irradiation in aqueous conditions delivering the product in good yields (70–89%). The use of a non-polar solvent resulted in the formation of side products like benzimidazole, indicating the importance of water as solvent in this protocol along with its high efficiency as absorber for microwave irradiation providing environmentally benign reaction conditions. The authors further extended the acid-catalyzed protocol for the synthesis of pentacyclic isoindole-fused furo[1,4]diazepines **26** using substituted 2-formylbenzoic acids **25**, phenylenediamine and tetronic acid with water as solvent (Scheme 8).

**Scheme 9:**
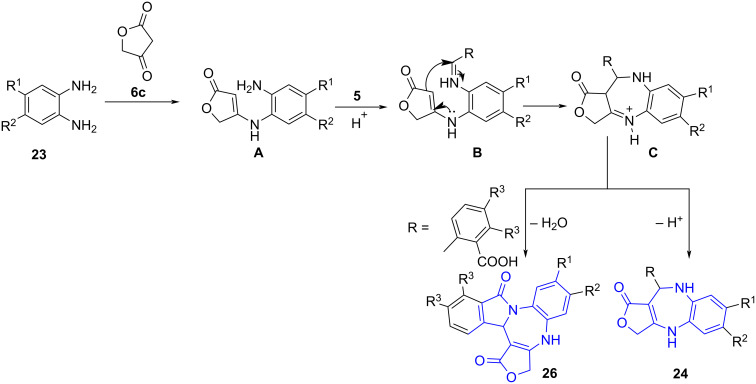
Proposed mechanism for benzoazulene-1-one synthesis.

The mechanism leading to the formation of the final product **24** and **26** involves an initial condensation between tetronic acid and benzene-1,2-diamine to give enaminone **A**. An intermediate **B** generated by the addition of aldehyde to enaminone **A** on intramolecular cyclization furnishes the final product **24** via **C**. Cyclic isoindole-fused furo[1,4]diazepines **26** were obtained by dehydration of the carbonyl group on the aromatic ring on treatment with an amino group. The authors attributed the high nucleophilicity of the amino group in the substrate **23** to control the regioselectivity of the reaction (Scheme 9).

### 3 Indoles

**Figure 3:**
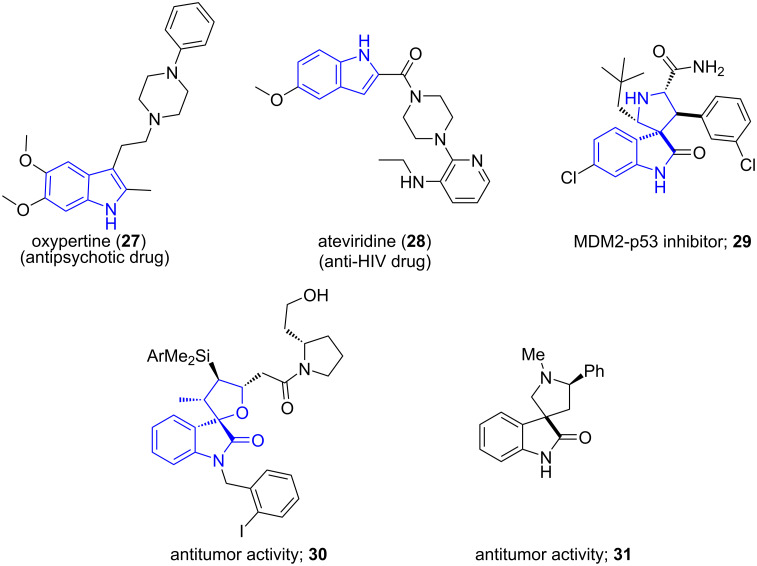
Indole-containing pharmacologically active molecules.

Indoles have a bicyclic structure consisting of a six-membered benzene ring fused with a five-membered nitrogen-containing pyrrole ring. Figure 3 depicts some of the marketed drugs structured around indole implying its pharmacological significance such as oxypertine (**27**), ateviridine (**28**) [47] and spirooxindole-based potent cytotoxic agents **29**, **30** and **31** [48].

**Scheme 10:**
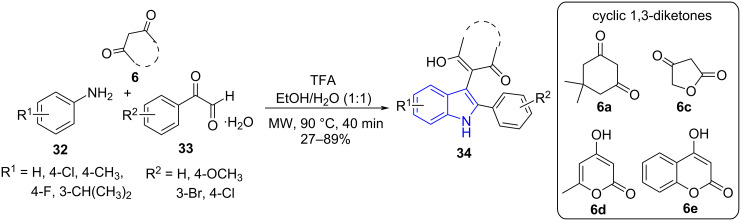
Synthesis of functionalized indoles.

**Scheme 11:**
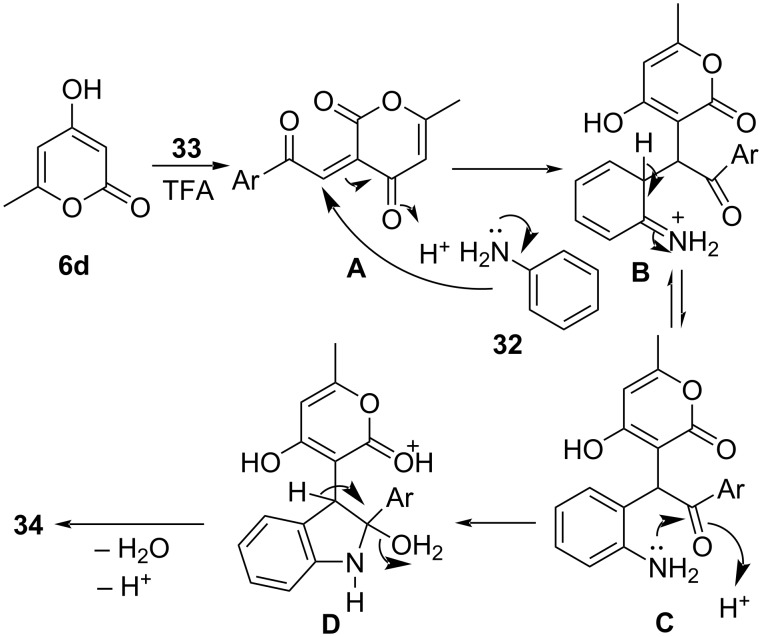
Plausible mechanism for the synthesis of functionalized indoles.

In 2017, Lin and co-workers [49] designed a TFA-catalyzed three-component reaction for the regioselective synthesis of 3-functionalized indoles **34** by employing amines **32**, arylglyoxal monohydrate **33** and cyclic 1,3-diketones **6** under microwave irradiation in the greener solvent system EtOH/H_2_O (Scheme 10). A plausible mechanism (Scheme 11) suggests a TFA-catalyzed Knoevenagel condensation between 4-hydroxy-6-methyl-2*H*-pyran-2-one and arylglyoxal to form intermediate **A**. Michael addition of amine to intermediate **A** gives **B** which further undergoes an intramolecular nucleophilic addition reaction to yield **C** which on cyclization and with subsequent loss of water from **D** produce the desired products **34**.

**Scheme 12:**
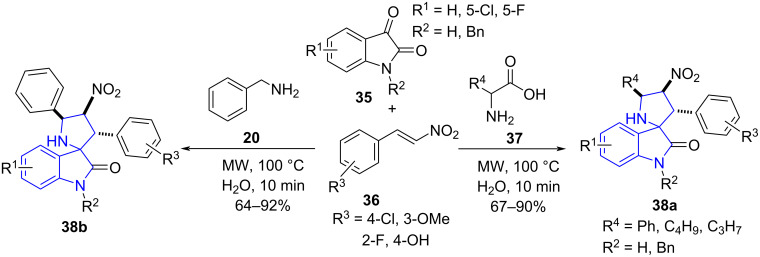
Synthesis of spirooxindoles.

Meshram and co-workers [50] demonstrated an aqueous phase, diastereoselective, multicomponent reaction involving substituted isatins **35**, β-nitrostyrene **36** and benzylamine (**20**) or α-amino acids **37** using microwave irradiation to afford a library of spirooxindoles **38** in good yields under catalyst-free conditions. Observations revealed that the conventional refluxing method produced only 10% of the desired product and brought microwave assistance to light. The synthesized molecules showed good antimicrobial activity against *Escherichia coli*, *Candida tropicalis*, *Staphylococcus aureus* and *Pseudomonas aeruginosa* (Scheme 12).

**Scheme 13:**
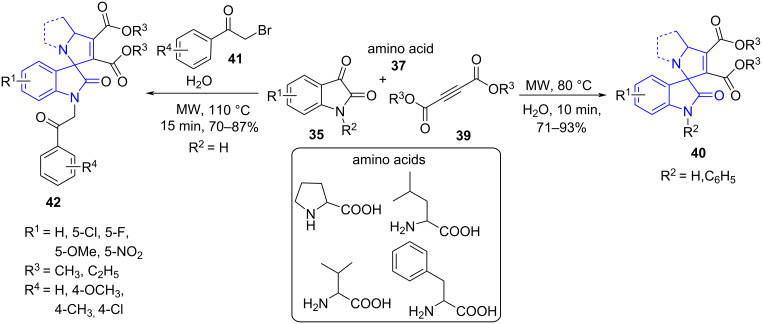
Synthesis of substituted spirooxindoles.

Similarly, the same group extended the work by illustrating [51] a three and four-component microwave-assisted base and catalyst-free reaction for the synthesis of substituted spirooxindoles **40**. The three-component reaction involved the reaction between substituted isatin **35**, but-2-ynedioates **39** and amino acids **37**. Likewise, the four-component reaction comprised of isatin **35**, but-2-ynedioates **39**, amino acids **37** and phenacyl bromides **41** to yield the *N*-acylated spirooxindoles **42** in good yields (Scheme 13). The reaction effectively explored the 1,3 dipolar compound generated with isatin and amino acids subjecting them to the potential dipolarophile but-2-ynedionates to deliver the target molecules. Both the reactions proceeded well in water aiding in greener synthesis of biologically active molecules. The synthesized molecules exhibited significant activity against human lung cancer cell line A549.

**Scheme 14:**
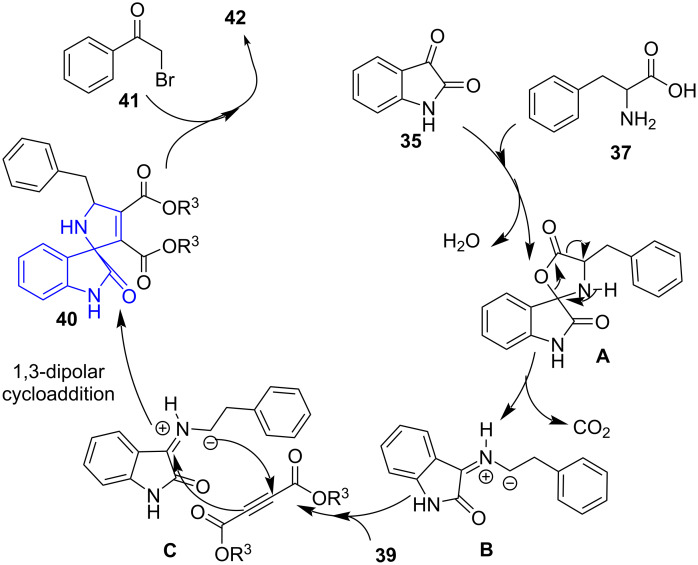
Plausible mechanism for the synthesis of substituted spirooxindoles.

A plausible mechanism shown in Scheme 14 explains the formation of azomethine ylide **B** by condensation of isatin with amino acid followed by release of a molecule of CO_2_ via **A**. The imine **B** undergoes 1,3-dipolar cycloaddition with the dipolarophiles **39**. The cyclization yields the desired product **40** of the three-component reaction whereas a further reaction with phenacyl bromide **41** results the product of the four-component reaction **42**.

**Scheme 15:**
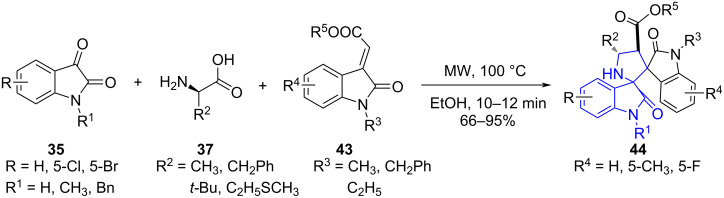
Synthesis of pyrrolidinyl spirooxindoles.

Recently, our group [52] efficiently employed the synergistic approach of MWA-MCR to deliver pyrrolidinyl spirooxindole **44**. The isatin **35**, primary amino acids **37** and 3-alkenyloxindole **43** were considered to be the building blocks united in ethanol as solvent (Scheme 15). The notable highlights of the described methodology are diastereoselective C–C and C–N bond formation, high yields, non-toxic product, and cost-effectiveness along with a greener approach.

**Scheme 16:**
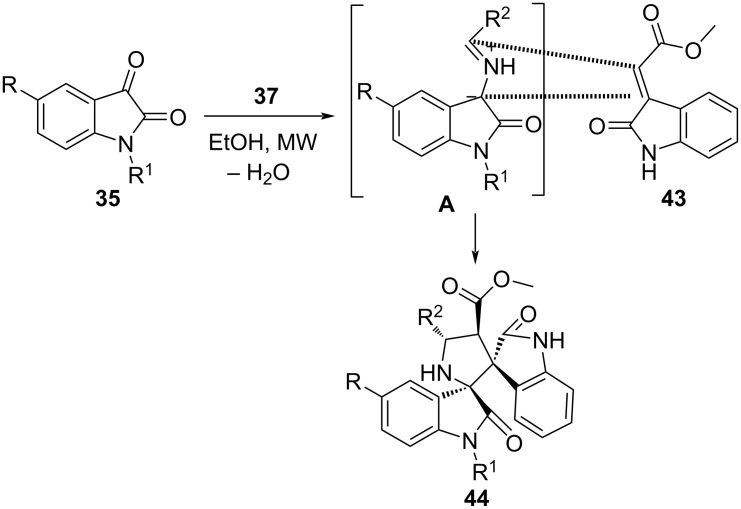
Proposed mechanism for pyrrolidinyl spirooxindoles.

The synthetic strategy introduces primary amines for 1,3-dipolar cycloaddition which is less explored due to the probability of competitive Strecker degradation over decarboxylation of azomethine ylides. The protocol reveals the efficiency of MW assisted reaction with reduced reaction time from 18 h to 12 min and enhanced the yield from 69% to 84% over the conventional protocol as observed during the study. The explored mechanism in Scheme 16 indicates an in situ *anti*-azomethine ylide (**A**) generation (between isatin and primary amine) favored due to steric hindrance in *syn*-ylide. The crucial step determines the route via ylide formation over the expected Strecker degradation. The azomethine ylide trapped by 3-alkenylindole undergoes 1,3-dipolar cycloaddition and led to the cycloadducts **44**.

### 4 Pyrans

**Figure 4:**
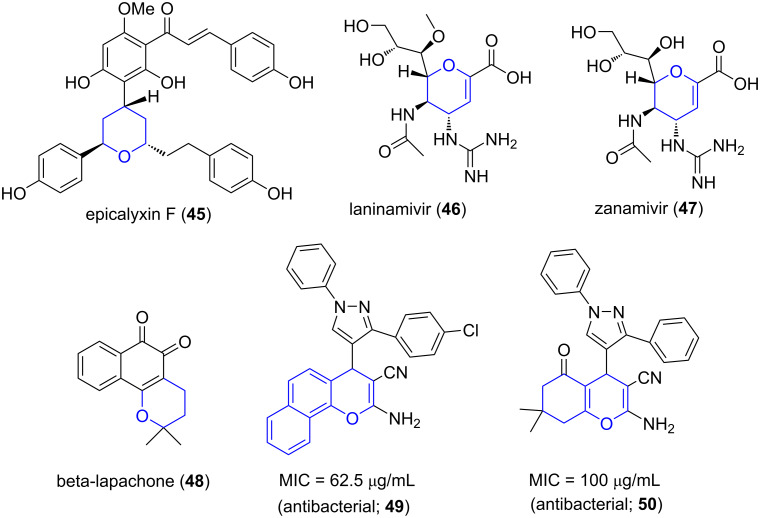
Pyran-containing biologically active molecules.

Pyran is a six-membered heterocyclic, non-aromatic ring, consisting of five carbon atoms and one oxygen atom with two double bonds. Numerous natural compounds containing pyrans and benzopyrans (fused pyrans) are identified. Epicalyxin (**45**) is used as an anticancer agent against human HT-1080 fibrosarcoma and murine 26-L5 carcinoma. Laninamivir (**46**) is a pyran-based drug used as a neuraminidase inhibitor and zanamivir (**47**) for prevention of influenza A and B. β-Lapachone (**48**) shows diverse biological activities like anticancer, antibacterial and anti-inflammatory activities [53]. Benzopyrans and naphthopyrans represent a class of fused pyrans that has been studied for antimicrobial effects (**49** and **50**, Figure 4) [54]. Therefore, researchers have quested upon generation of pyrans and benzopyrans employing MCR powered by microwave assistance.

**Scheme 17:**
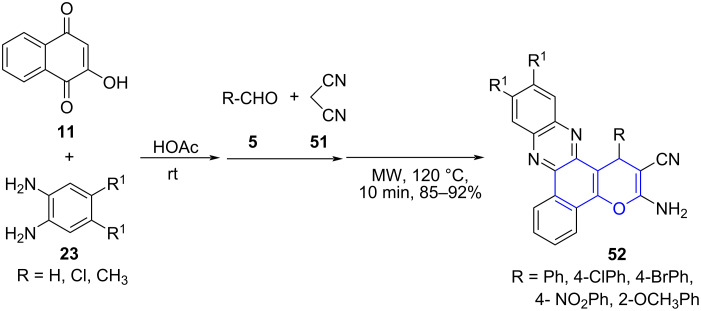
Synthesis of functionalized benzopyrans.

For instance, Tu and co-workers [55] reported a one-pot two-step tandem procedure subjecting phenylenediamine **23**, 2-hydroxynaphthalene-1,4-diones (**11**), aldehyde **5** with malononitrile (**51**) in presence of acetic acid under microwave irradiation for the synthesis of highly functionalized benzopyrans **52**. The method was successfully employed for the construction of chromene and phenazine motifs exhibiting the applicability of the protocol to engender diverse chemical entities (Scheme 17). The harsh reaction conditions with longer reaction time and limited substrate scope highlights the importance of the above mentioned strategy to obtain such fused molecules [56].

**Scheme 18:**
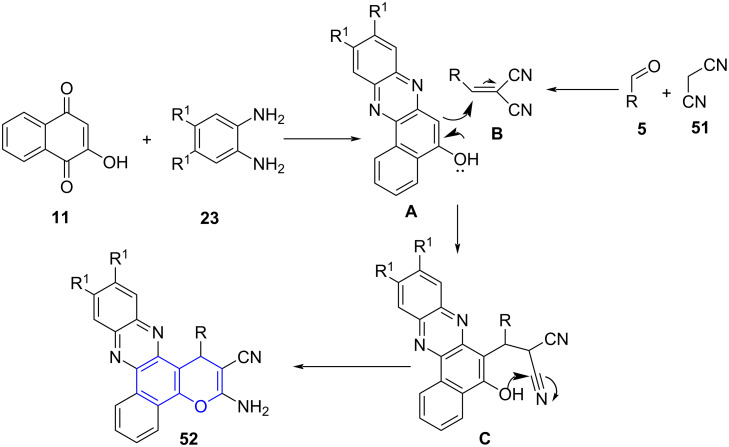
Plausible mechanism for synthesis of benzopyran.

A detailed mechanism was proposed, with the initial formation of benzo[*a*]phenazin-5-ol **A** through condensation of diamine **23** and 2-hydroxynaphthalene-1,4-dione (**11**). A simultaneous condensation between malononitrile and aldehyde afforded 2-benzylidenemalononitrile **B** which on Michael addition with condensed intermediate **A** yields intermediate **C**, to undergo cyclization and resulted in the desired products (**52**, Scheme 18).

**Scheme 19:**
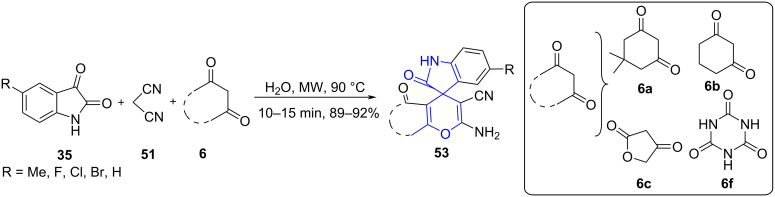
Synthesis of indoline-spiro-fused pyran derivatives.

The synthesis of indoline-spiro fused pyran derivatives **53** was reported by Jiang and co-workers [57] employing a multicomponent reaction between substituted isatins **35**, cyclic 1,3-diketones **6** and malononitrile (**51**) in an aqueous medium without any catalyst. Reaction diversity was examined by using different 1,3-diketones and isatins (Scheme 19). Products obtained from non-chromatographic techniques such as filtration proved the versatility of the strategy.

**Scheme 20:**
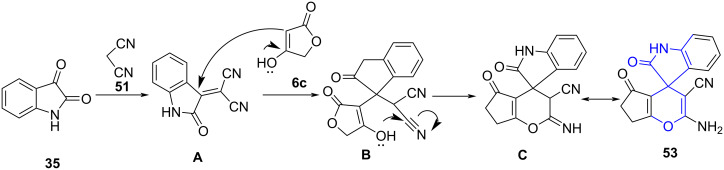
Proposed mechanism for indoline-spiro-fused pyran.

A rationale mechanism for the synthesis of **53** was described in Scheme 20. Incipiently, a fast Knoevenagel condensation between isatin and malononitrile produced isatylidene malononitrile derivative **A**. This intermediate **A** undergoes Michael addition with tetronic acid to afford an intermediate **B**. Ultimately, the cycloaddition of the hydroxy group to the cyano group afforded the desired product **53** via **C**.

**Scheme 21:**
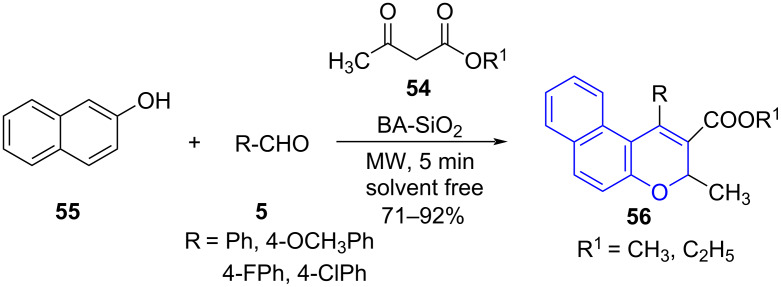
Synthesis of substituted naphthopyrans.

Meanwhile, Nepali and co-workers [58] reported the potential of naphthopyrans as non-purine xanthine oxidase inhibitors. They explored a silicated fluoroboric acid-catalyzed three-component cycloaddition involving acyclic 1,3-diketones **54**, β-naphthol (**55**) and aldehyde **5** for the synthesis of substituted naphthopyrans **56** under microwave irradiation under solvent-free conditions. The library of compounds proved to be active as xanthine oxidase inhibitors with the most potent molecule showcasing IC_50_ = 4 μM (Scheme 21).

### 5 Pyrroles

**Figure 5:**
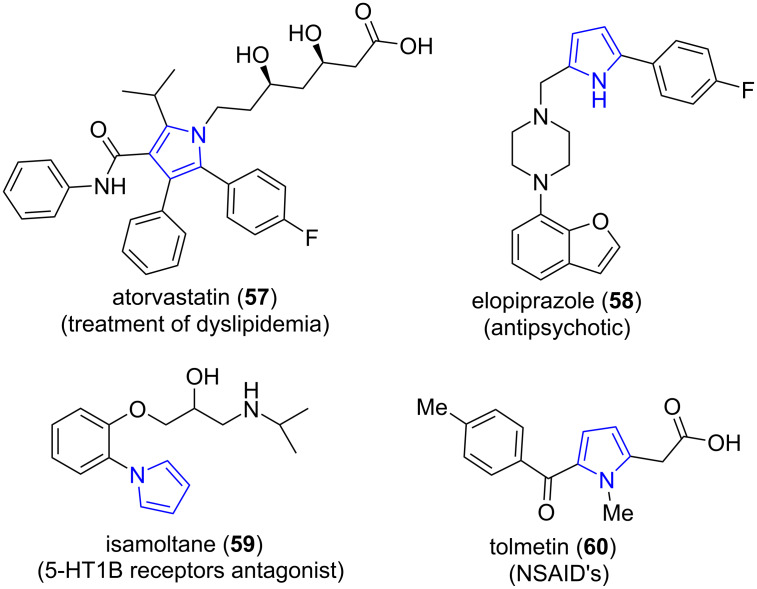
Marketed drugs with pyrrole ring.

Pyrroles are five-membered heterocycles consisting of four carbon atoms and a nitrogen atom. The pyrrole ring is found to be abundant in a plethora of lead molecules and marketed drugs like atorvastatin (**57**), elopiprazole (**58**), isamoltane (**59**) and tolmetin (**60**, Figure 5) [59–60].

**Scheme 22:**
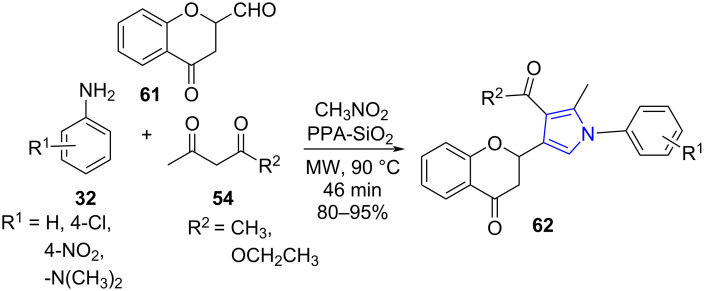
Synthesis of tetra-substituted pyrroles.

The diverse pharmacological activities of pyrroles enlivened Kumar and co-workers [61] to report a facile and eco-friendly microwave-assisted four-component reaction involving chromene-aldehyde (**61**), amines **32**, acyclic 1,3-diketones **54** and nitromethane using silica-gel-supported polyphoshoric acid as catalyst under neat conditions for the synthesis of tetra-substituted pyrroles **62**. A comparative study of the protocol employing the conventional and microwave approach proved the microwave strategy to be advantageous with enhanced yield from 87–95% in reduced time (3 h to 46 min). The parameters were successful in overcoming the drawbacks such as functional group compatibility, regiospecifity, multi-step procedure etc. suffered by traditional methods [62–63]. The catalyst offered recovery and reusability up to five successive runs with excellent yields (86% to 95%). The approach paved a new way to solid-support-mediated MWA-MCR using a heterocatalyst (Scheme 22).

**Scheme 23:**
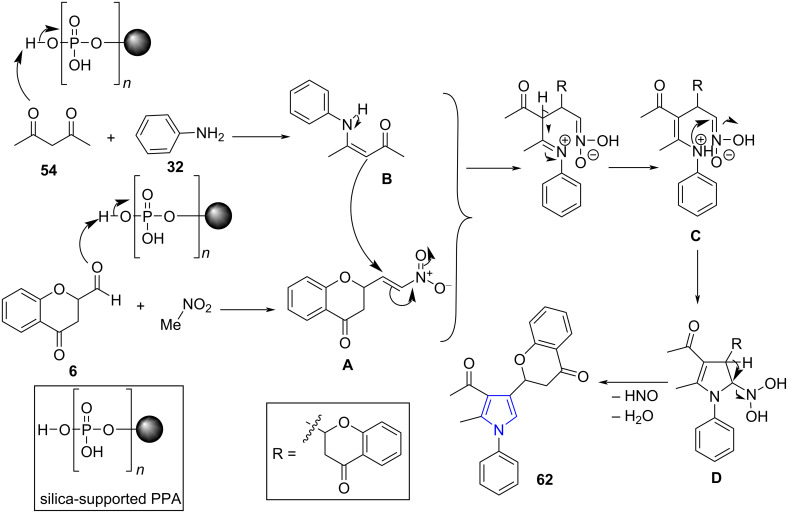
Mechanism for silica-supported PPA-SiO_2_-catalyzed pyrrole synthesis.

A possible mechanism suggested by authors proceeded via a Michael addition between nitrostyrene adducts **A** and β-keto enamine **B** generated in situ consequently undergoes cyclization **C** and dehydration **D** to afford the desired product **62**. PPA-SiO_2_ accelerates the reaction by enhancing the electrophilicity of the 1,3-diketones and the aldehydes by increasing the rate of generation of the β-enaminocarbonyl and nitrostyrene intermediates. Activation of Michael addition followed cyclization was catalyzed by silica-supported PPA-SiO_2_ (Scheme 23).

**Scheme 24:**
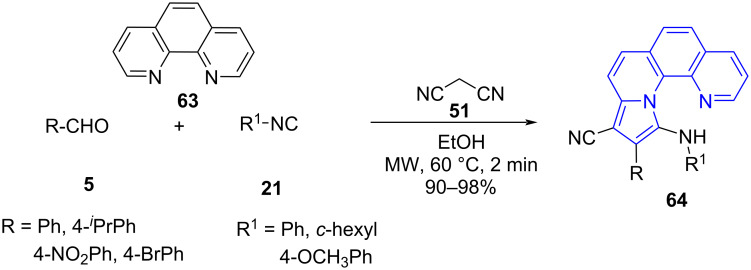
Synthesis of pyrrolo[1,10]-phenanthrolines.

Fused pyrroles have also been constructed by exploring the utility of a multicomponent reaction coupled with microwave irradiation. One such demonstration was reported by Padmini and co-workers [64] wherein a four-component reaction between substituted aldehydes **5**, phenanthroline (**63**), malononitrile (**51**) and isocyanides **21** afforded pyrrolo[1,10]-phenanthrolines **64** in ethanol as a solvent with excellent yields. The conventional approach delivered the desired product in 62% yield after 6 h which reveals the efficiency of microwaves in increasing the yield and reducing the reaction time. The studies for the anticancer activity of the synthesized molecules revealed them to be more potent than the standard doxorubicin against AGS cancer cell lines along with good antimicrobial activity (Scheme 24).

**Scheme 25:**
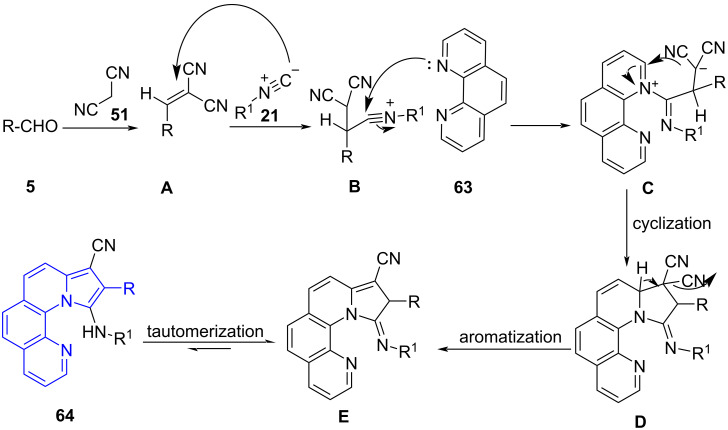
Proposed mechanism for pyrrolo[1,10]-phenanthrolines.

The proposed mechanism (Scheme 25) involved a Knoevenagel condensation between aldehyde **5** and malononitrile (**51**) to form arylidene intermediate **A**. Then **A** reacts with isocyanide **21** to produce intermediate **B** which coordinates with 1,10-phenanthrolines and affords intermediate **C**. A subsequent cyclization **D** and aromatization **E** with loss of HCN yield the desired products **64**. The lower yields in case of aliphatic isocyanides were reasoned with its low nucleophilicity losing the competition with aryledenemalononitrile **A** in the reaction with phenanthroline.

### 6 Pyrimidines/fused pyrimidines

#### 6.1 Pyrimidines

**Figure 6:**
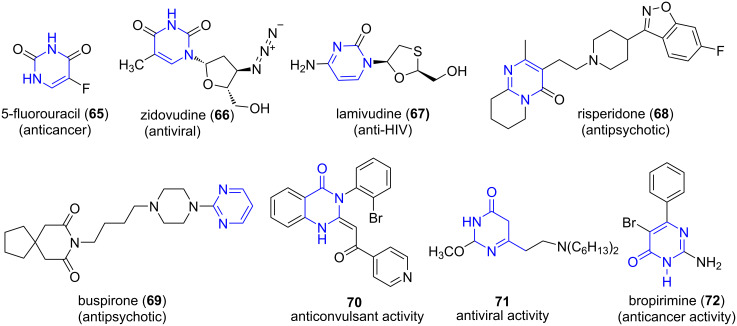
Marketed drugs and molecules containing pyrimidine and pyrimidinones skeletons.

Pyrimidines are six-membered aromatic heterocycles containing two nitrogen atoms at positions 1 and 3. These are an important class of compounds depicting a wide range of biological activities such as COX inhibitors, anti-inflammatory, anticancer, analgesic, etc. They form a major structural constituent of biomolecules like DNA and significant drugs like fluorouracil (**65**), zidovudine (**66**), lamivudine (**67**), risperidone (**68**), and buspirone (**69**, Figure 6) [65]. The biological importance of pyrimidinones like anticonvulsant (**70**)**,** antiviral (**71**) and anticancer activities (**72**, Figure 6) [66–69] prompted chemists to develop newer methodologies for the synthesis of pyrimidinones with atom economy and high yields.

The Biginelli reaction is one of the frequently employed MCRs for the synthesis of dihydropyrimidinones. The classical Biginelli reaction suffers from drawbacks such as harsh reaction conditions, longer reaction time and low yields [70]. Several attempts have been made to improve the reaction conditions using various catalyst/reagents, ionic liquids etc. [71–72].

**Scheme 26:**
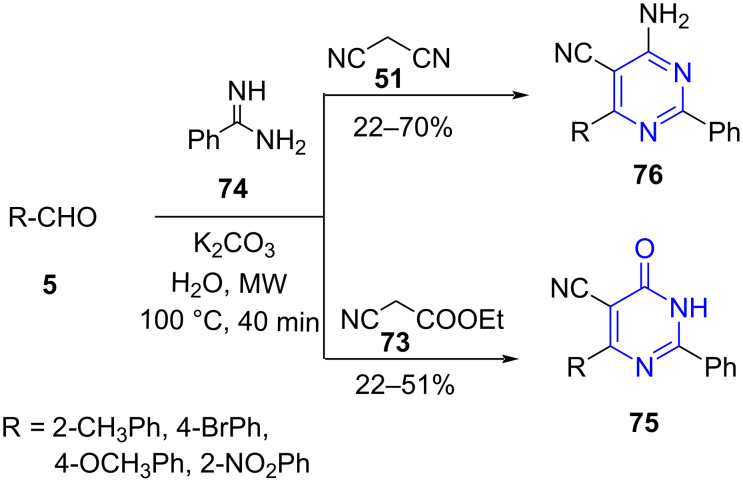
MWA-MCR pyrimidinone synthesis.

Contributing to this need, dos Anjos et al. [73] reported a base-catalyzed three-component reaction between aromatic aldehydes **5,** ethyl cyanoacetate (**73**) (active methylene group) and benzamidine (**74**) in aqueous media for the construction of substituted pyrimidinones **75** under microwave irradiation (Scheme 26). The study of the protocol on a conventional system directed a reduced yield of mere 18% in 16 h. A slight variation to the protocol with malononitrile (**51**) as the active methylene compound affords a series of substituted 4-aminopyrimidines **76** in moderate yields. The efficacy of the synthesized dihydropyrimidinones as antinociceptive was also established.

**Scheme 27:**
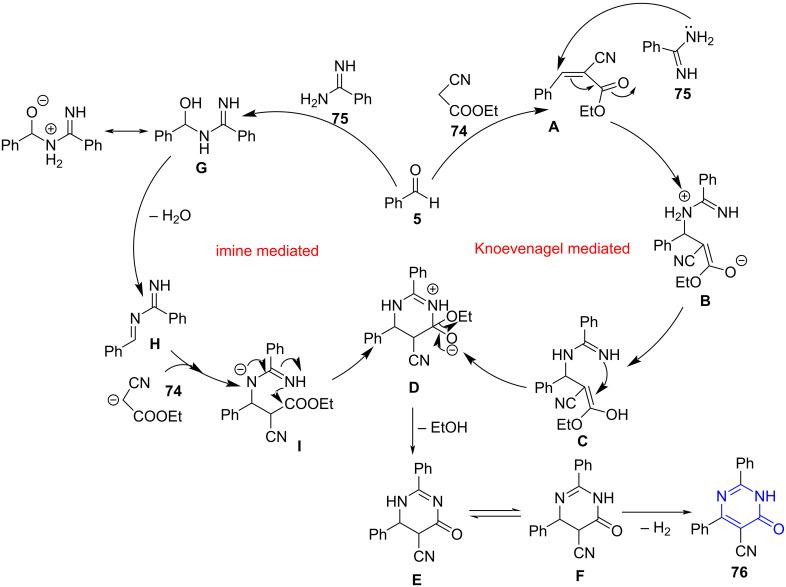
Two proposed mechanisms for pyrimidinone synthesis.

The authors proposed two different mechanisms in which the first mechanism involved two subsequent reactions. The first one being a Knoevenagel condensation between aromatic aldehyde and ethyl cyanoacetate to yield a Knoevenagel intermediate **A** which upon subsequent reaction with benzamidine, forms Michael adduct **B**. A consecutive ring closure yields the desired product **75** aided by the attack of nitrogen lone pair in Michael’s adduct **C** via a sequential ethanol elimination (**E**) from **D** followed by aerial oxidation of intermediate **F**. Another proposed mechanism follows the formation of imine derivative **G** produced by the reaction between aldehyde and amidine. The imine **G** thereby reacts with ethyl cyanoacetate to result in intermediate **I**, which on intramolecular cyclization leads to **D.** The remaining pathway pursues same mechanism as the first one (Scheme 27).

**Scheme 28:**
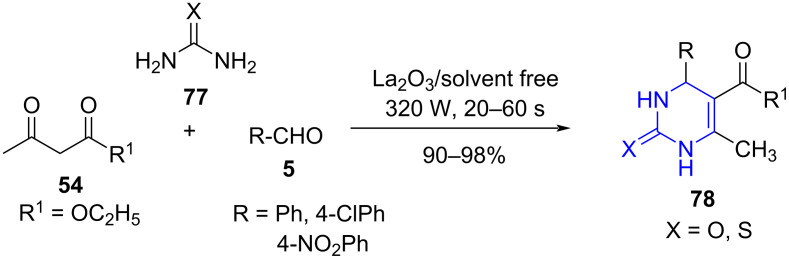
MWA multicomponent synthesis of dihydropyrimidinones.

Later in 2016, Gopalakrishnan and co-workers [74] demonstrated the construction of dihydropyrimidinones **78** utilizing a three-component reaction of acyclic 1,3-diketones **54**, urea/thiourea (**77**) and aldehyde **5** exploring La_2_O_3_ as catalyst under microwave irradiation under solvent-free conditions with good functional group tolerance and excellent yields (Scheme 28). The reaction failed to produce the desired product at room temperature even after extended period of time. A comparative analysis of the strategy with different catalyst under refluxing conditions surfaced the efficiency of microwave in reducing the time from hours to seconds and increasing the yield considerably.

**Scheme 29:**
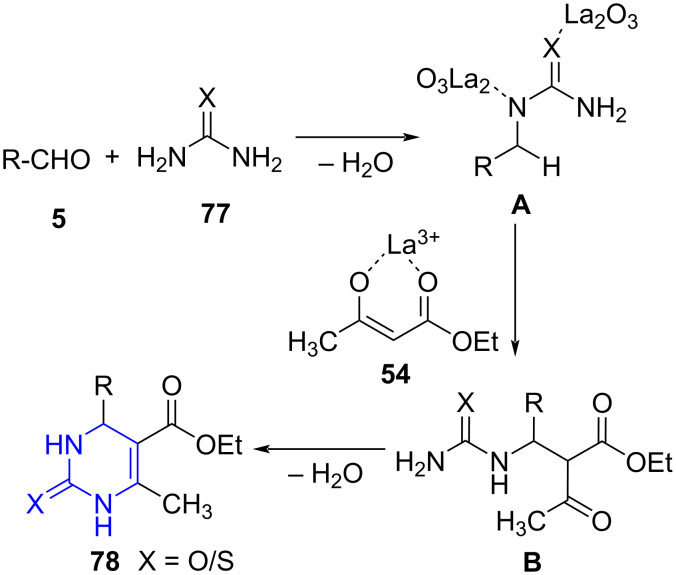
Proposed mechanism for dihydropyrimidinones.

The postulated mechanism indicates the formation of acylimine **A** from the lanthanum oxide-catalyzed reaction of aldehyde and **77**. Further, addition of acyclic 1,3-diketone ester enolate to acylamine **A** form **B** which upon subsequent cyclization and dehydration resulted in the formation of desired products **78** (Scheme 29).

#### 6.2 Fused pyrimidines

**Figure 7:**
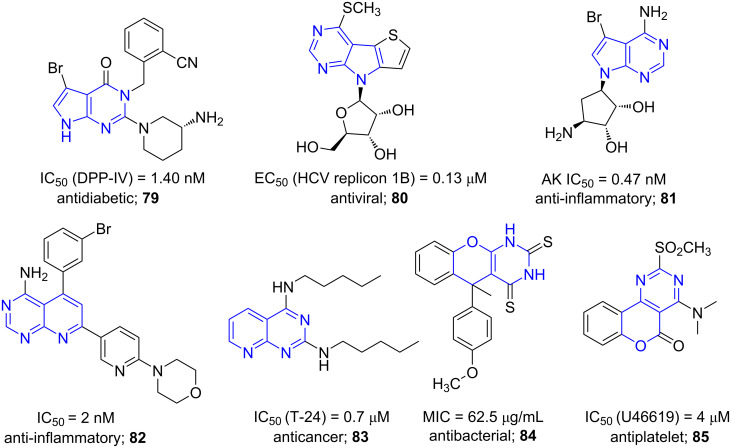
Biologically active fused pyrimidines.

Fused pyrimidines represent an important class of heterocycles with potential biological activities such as antidiabetic (**79**), antiviral (**80**), anti-inflammatory (**81**, **82**), anticancer (**83**), antibacterial (**84**) and antiplatelet (**85**) [75–79] with an advantage of the synergistic action of the two pharmacophores fused (Figure 7).

**Scheme 30:**
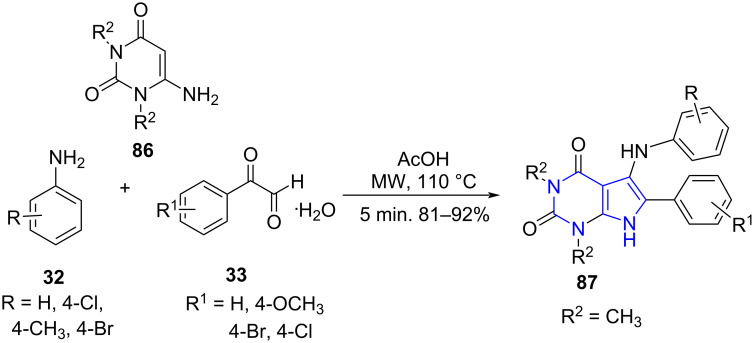
MWA- MCR for the synthesis of pyrrolo[2,3-*d*]pyrimidines.

**6.2.1 Pyrrolo[2,3-*d*]pyrimidines:** Bhuyan and co-workers [80] reported an efficient MWA three-component reaction between *N,N*-disubstituted-6-aminouracil **86**, arylglyoxal monohydrate **33**, and amines **32** in AcOH resulting in the synthesis of 5-arylaminopyrrolo[2,3-*d*]pyrimidines **87** in good to excellent yields (Scheme 30). The dual-use of acetic acid as a catalyst and solvent along with simple filtration and a recrystallization procedure to obtain pure products adds advantage over the other reported protocols [81–82].

**Scheme 31:**
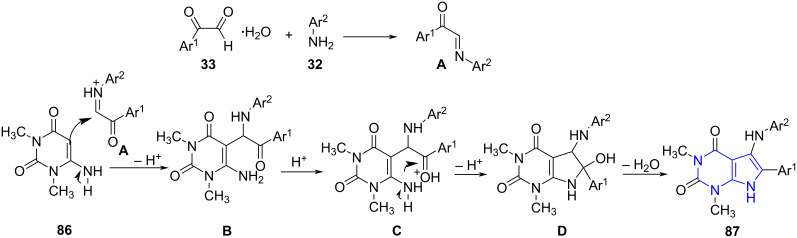
Proposed mechanism for pyrrolo[2,3-*d*]pyrimidines.

A rational mechanism describes the synthesis by the formation of an intermediate **A** from the condensation between compounds **33** and **32** in presence of an acid undergoing nucleophilic addition with **93** resulting in intermediate **B**. This intermediate undergoes an intramolecular cyclization **C** aided by an acid to give intermediate **D** which on the loss of a water molecule yields the products **87** (Scheme 31).

**Scheme 32:**
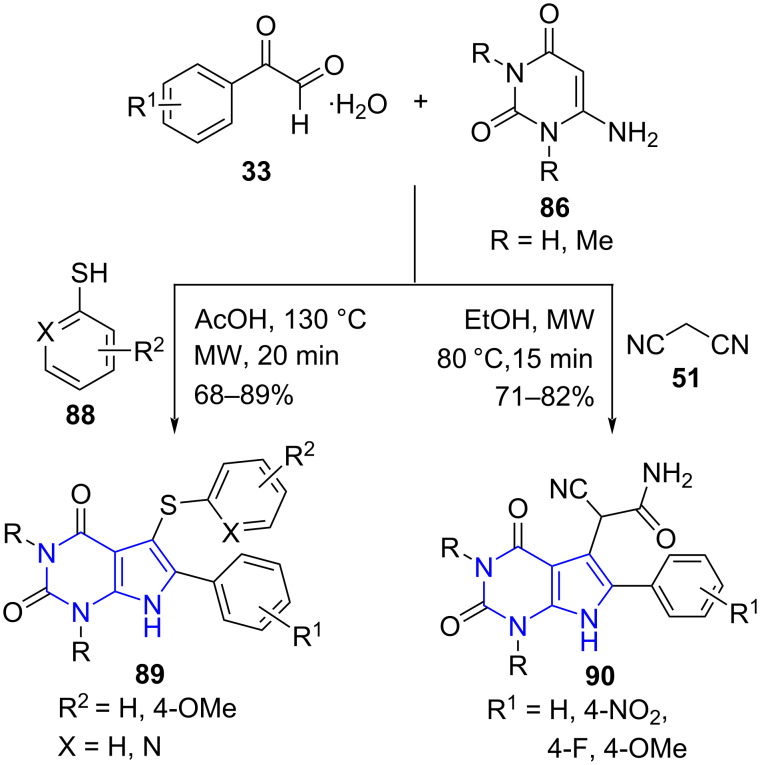
Synthesis of substituted pyrrolo[2,3-*d*]pyrimidine-2,4-diones.

Choudhury and co-workers [83] disclosed a three-component reaction of substituted arylglyoxal monohydrate **33,** 6-amino-1,3-disubstituted uracil **86** and substituted thiols **88** under microwave conditions using acetic acid as a solvent to successfully furnish 5,6-disubstituted pyrrolo[2,3-*d*]pyrimidine-2,4-diones **89**. Similarly, excellent yields were obtained when the thiol was replaced by malononitrile (**51**) even in the absence of catalyst or any promoter. The malononitrile undergoes hydrolysis forming an amide, thus giving rise to a series of pyrrolo[2,3-*d*]pyrimidine-2,4-diones acetamides **90** under microwave irradiation (Scheme 32). An interesting observation by the authors surfaced that the conventional approach to the malononitrile protocol delivered comparable yields in longer reaction time (5–8 h). A gram-scale attempt under microwave conditions delivered the desired product in better yields than the reflux strategy.

**Scheme 33:**
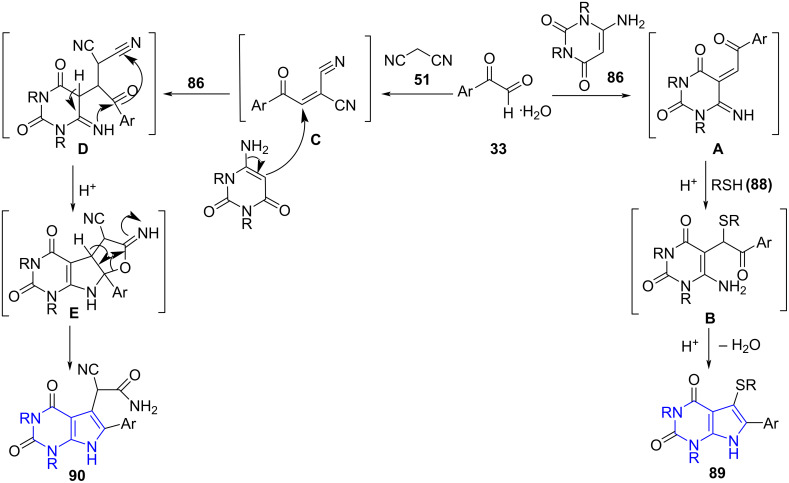
Probable pathway for pyrrolo[2,3-*d*]pyrimidine-2,4-diones.

Scheme 33 depicts the most probable pathway for the desired products **89** and **90**. Initially, an acid-catalyzed reaction between arylglyoxal and amino uracil yields intermediate **A**. Nucleophilic addition of thiol to intermediate **A** results in the formation of intermediate **B**. An intramolecular cyclization followed by dehydration of intermediate **B** ultimately produces **89**. On the other hand, intermediate **C** is formed by the Knoevenagel condensation between arylglyoxal and malononitrile. This is followed by the Michael addition of aminouracil to intermediate **C** to give **D**. Finally, desired product **90** is formed by intramolecular cyclization of intermediate **D** and subsequent rearrangement of **E**.

**Scheme 34:**
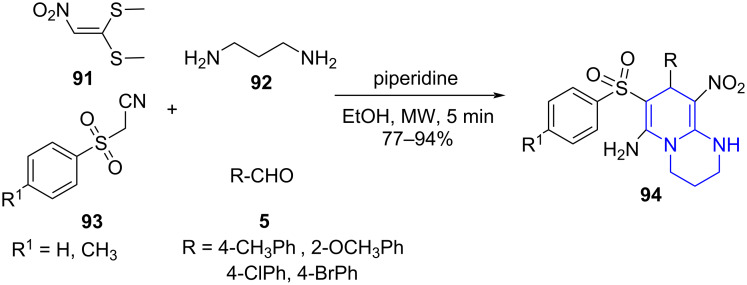
Synthesis of pyridopyrimidines.

**6.2.2 Pyridopyrimidines:** Zhang and co-workers [84] proposed a synthetic route for the construction of substituted pyridopyrimidines **94** utilizing a piperidine-catalyzed microwave-assisted four-component reaction by employing 1,1-dimethylthio-2-nitroethylene (**91**), 1,3-propanediamine (**92**), phenylsulfonyl acetonitrile **93** and aldehyde **5** in ethanol as solvent (Scheme 34). The crucial role of time was realized when the yield of the reaction increased with time up to 5 min a further increase in time decreased the yield, attributed to the formation of byproduct and decomposition of product.

**Scheme 35:**
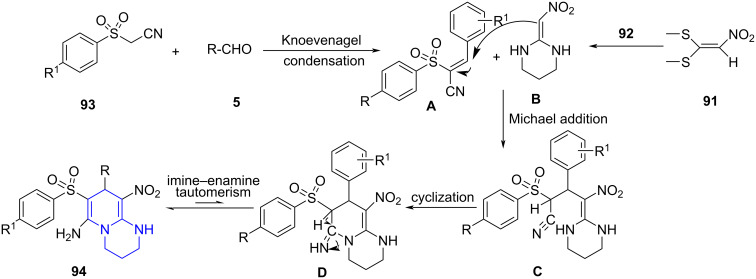
Plausible mechanism for the synthesis of pyridopyrimidines.

A plausible mechanism proposed by the authors indicates the Knoevenagel condensation between **5** and **93** to form adduct **A** and undergo an aza-ene reaction with 2-(nitromethylene)hexahydropyrimidine **B** (obtained by reaction between **92** and **91**) resulting in intermediate **C**. The nucleophilic addition of secondary amino to cyano group affords an intermediate **D** engaging in imine–enamine tautomerism and finally leads to desired products **94** (Scheme 35).

**Scheme 36:**
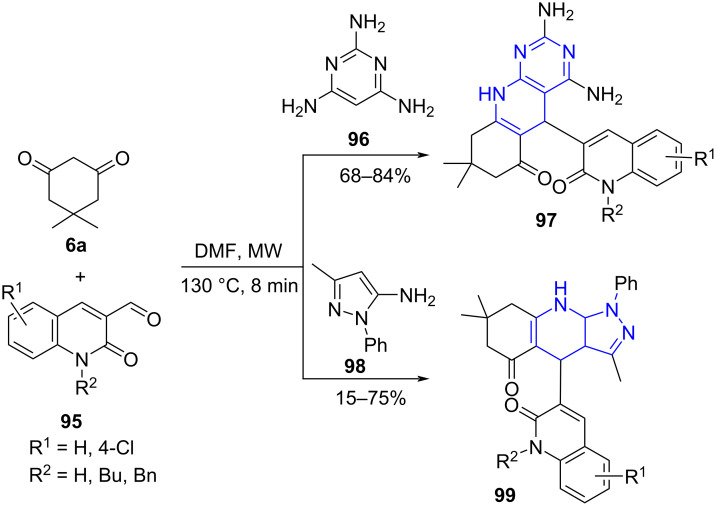
Synthesis of dihydropyridopyrimidine and dihydropyrazolopyridine.

Abonia and co-workers [85] established a catalyst-free construction of quinoline-based pyridopyridines **97** by employing a microwave-assisted three-component reaction of 3-formyl-2-oxoquinoline derivatives **95**, 2,4,6-triaminopyrimidine (**96**) and a cyclic 1,3-diketone such as dimedone (**6a**) in DMF. The resulting products were obtained in moderate to good yields. The authors observed the formation of pyrazolopyridine under conventional heating in lower yield (38%) with extended reaction time (20 h). Interestingly, the replacement of triaminopyrimidine with substituted aminopyrazoles **98** resulted in functionalized dihydro-1*H*-pyrazolo[3,4-*b*]pyridines **99** under the same conditions. Moreover, the reaction preceded well even with other 1,3-diketones along with primary heterocyclic amines (Scheme 36). The modest yields of **99** compared to **97** were reasoned with the decomposition of the amines. Suprisingly, the adaptation of conventional strategy delivered the aromatized product in better yields (62–75%). The increased yield was attributed to the lower decomposition observed for the starting material amines. The authors proposed that the final aromatized product was derived from the initial formation of the dihydro derivative **99**, followed by aromatization under the described conditions. The preliminary in vitro antitumor studies of the compounds displayed low to moderate activity.

**Scheme 37:**
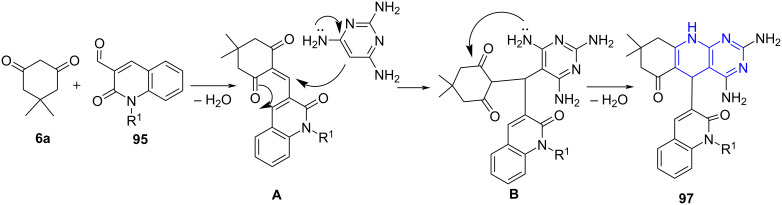
Proposed mechanism for the formation of dihydropyridopyrimidine.

Scheme 37 depicts the mechanism wherein an initial in situ generation of α,β-unsaturated intermediate **A** occur due to the Knoevenagel condensation between ketone and formylquinoline. The amine and the intermediate **A** undergo Michael addition furnishing the keto-amine **B**. Further, an intramolecular cyclization with the attack of the amino group onto the carbonyl functionality with subsequent elimination of a water molecule results in the desired product **97**.

**Scheme 38:**
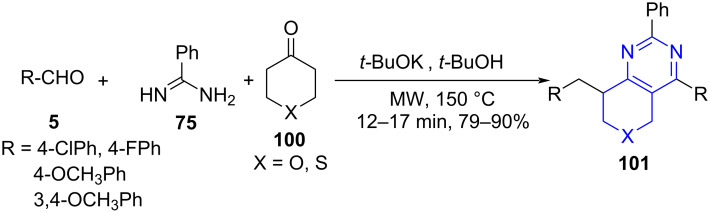
Synthesis of thiopyrano[4,3-*d*]pyrimidines.

**6.2.3 Thiopyrano-, pyrano[4,3-*d*]pyrimidines:** Jiang and co-workers [86] proposed a three-component reaction involving aldehydes **5,** tetrahydrothiopyran-4-ones **100** and amidines **75** under microwave irradiation using *t-*BuOH as solvent and *t-*BuOK as a base. This reaction provided easy access to the synthesis of thiopyranopyrimidines **101** with regiospecific positioning of the benzyl group at 8-position as presented in Scheme 38. Simple filtration affords the desired products with purity.

**Scheme 39:**
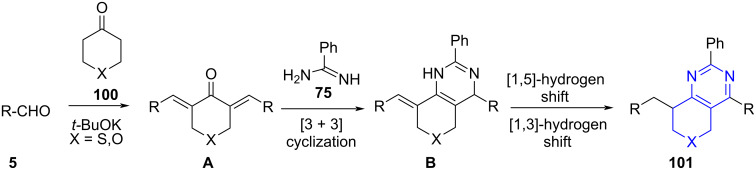
Plausible mechanism for the synthesis of thiopyrano[4,3-*d*]pyrimidines.

A plausible mechanism suggests the formation of 2,6-dibenzylidene heterocyclic ketones **A** by the condensation of aromatic aldehydes and heterocyclic ketones followed by a [3 + 3] cycloaddition between **A** and amidine giving off the intermediate **B**, which undergoes 1,5-hydrogen transfer followed by 1,3-hydrogen transfer to give the final products (**101**, Scheme 39).

**Scheme 40:**
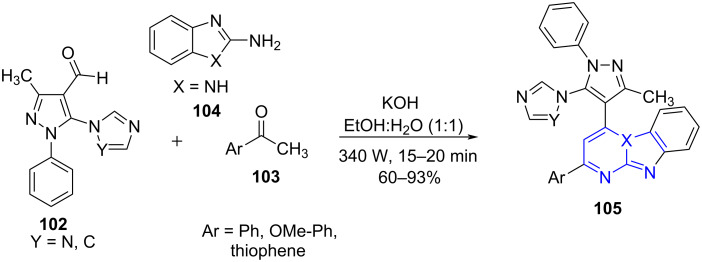
Synthesis of decorated imidazopyrimidines.

**6.2.4 Imidazo(1,2-*a*)pyrimidine:** The generation of imidazo-heterocycles has been a daunting challenge for the chemist due to the harsh condition requirements, such as multi-step protocol, high temperature and longer reaction time [87–89]. Overcoming these synthetic barriers, Patel and co-workers [90] developed an efficient microwave-assisted protocol for the construction of imidazopyrimidine clubbed pyrazoles **105**. The one-pot one step/two step approach by the authors employed a KOH-mediated reaction of 4-carbaldehyde pyrazoles **102**, acetophenones **103** and 2-aminobenzimidazole (**104**) in a greener solvent mixture of ethanol/water (1:1) under microwave irradiation at 340 W (Scheme 40). The conventional approach delivered the desired products in a two-step procedure with prolonged reaction time (28 h) advocating the efficiency of microwave technology. The protocol was used to design densely diversified imidazopyrimidines, which were further studied for their antimicrobial, antituberculosis and antimalarial effects.

**Scheme 41:**
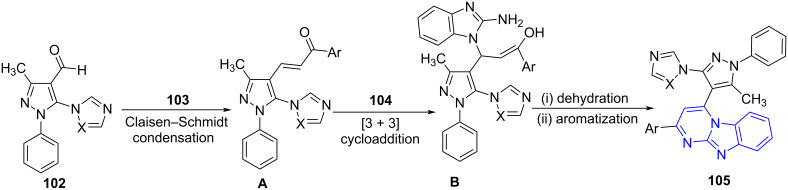
Proposed mechanism for imidazopyrimidine synthesis.

Scheme 41 demonstrates the mechanism involving a direct Claisen–Schmidt condensation to intermediate **A** followed by a sequential [3 + 3] cycloaddition with **104** yields **B**. Finally, dehydration and hydrogen removal from **B** furnished the desired products **105** in good to moderate yields.

### 7 Purines

**Figure 8:**
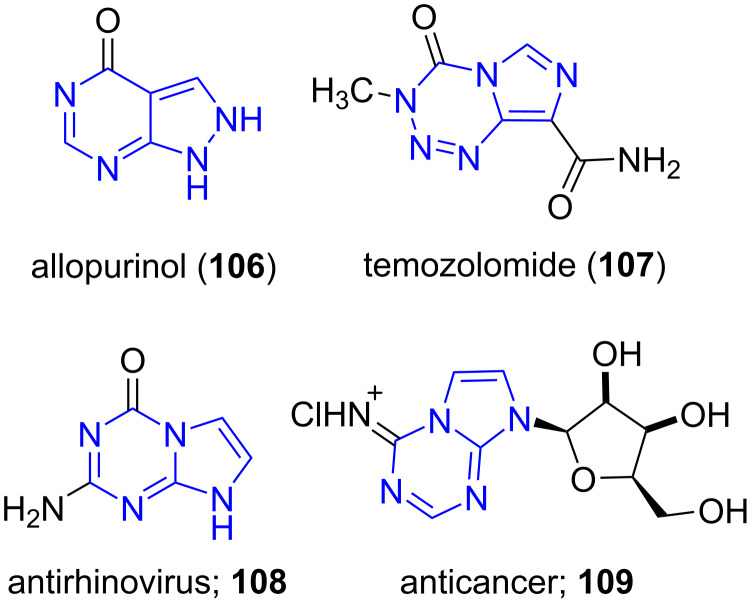
Pharmacologically active molecules containing purine bases.

Purines are categorized as heterocyclic aromatic compounds, consisting of a pyrimidine ring fused to an imidazole ring. Adenine and guanine are purine nitrogenous bases found in nucleic acids. Utilizing purine analogs as isosteres are well-thought-out as an important approach in medicinal chemistry and in drug discovery domains [91–92]. Purine scaffolds, such as allopurinol (**106**) used as the first choice of drug in gout therapy and temozolomide (**107**) used in the treatment of brain cancer are well-known examples [93–94]. Among the purine scaffolds, 5-aza-9-deazapurine and 5-azapurine have been identified as a favorable skeleton for the construction of new compounds such as **108** and **109** (Figure 8) [95–96].

**Scheme 42:**
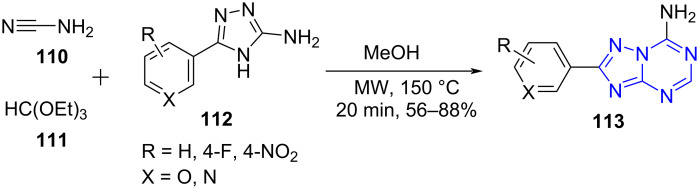
Synthesis of aza-adenines.

Considering this fact, Dolzhenko and co-workers [97] reported the first microwave-assisted multicomponent strategy for the regioselective construction of substituted 5-aza-adenines **113** using cyanamide (**110**), triethyl orthoformate (**111**) and 5-amino-1,2,4-triazoles **112** as structural units with methanol as solvent (Scheme 42). Simple filtration with no product isomer formation gives this protocol an edge over the other traditional methods [98–99]. The conventional method produced the target molecule in a trace amount (1.5%). Addition of TMSCl to this approach resulted in 2.5% yield of the desired product. On the contrary in the presence of microwave irradiation the regioselctive 5-aza-adenine was afforded in 65% yield within 20 min in the absence of TMSCl. This indicates the importance of microwave in the construction of such pharmacologically relevant molecules under benign conditions.

**Scheme 43:**
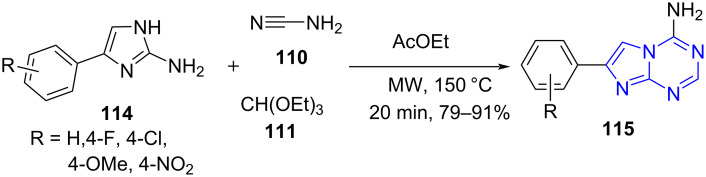
Synthesis of 5-*aza*-7-deazapurines.

Similarly, the same group [100] explored an one-pot three-component reaction involving cyanamide (**110**), 2-amino-4-phenylimidazole **114** and triethyl orthoformate (**111**) using ethyl acetate as a solvent for the exclusive synthesis of 5-aza-7-deaza-adenines **115** over the other regioisomer (**A**) in good to excellent yields (Scheme 43). A comparative study with the conventional approach produced the desired product in 13% yield with an elongated reaction time of 24 h. A scale protocol adapted with same reaction conditions afforded the product with a better yield of 92% supporting the scale-up strategy with microwaves. The protocol provided an easy admittance to 5-aza-7-deazapurine molecules used as antiviral and cytotoxic agents [101].

**Scheme 44:**
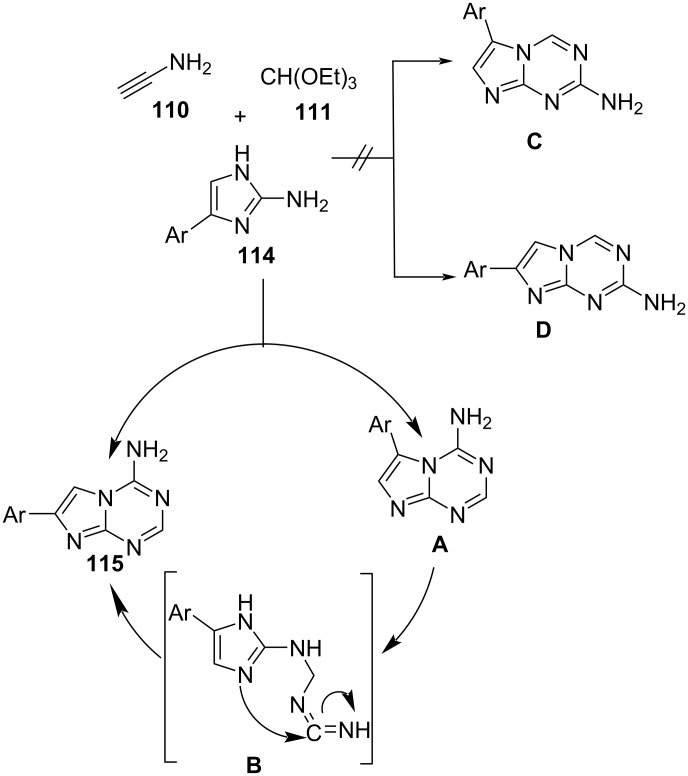
Proposed mechanism for deazapurines synthesis.

The potential rearrangement explained the regioselectivity during ring closure as depicted in Scheme 44. Theoretically, two regioisomeric pairs of adenine (**115**, **A**) and isoadenine are possible (**C**, **D**) (Scheme 44). However, using the multicomponent approach one product, 4-amino-7-arylimidazo[1,2-*a*][1,3,5]triazines **115**, could be only obtained. The regioisomer **A** being less stable due to steric hindrance between the amino and the aryl group rearranges to give desired product **115**. The mechanism involved in the formation of intermediate **B** is similar to the mechanism proposed for amino-1,3,5-triazine ring rearrangement [102] in an analogous heterocyclic system.

### 8 Pyridines/fused pyridines

#### 8.1 Pyridines

**Figure 9:**
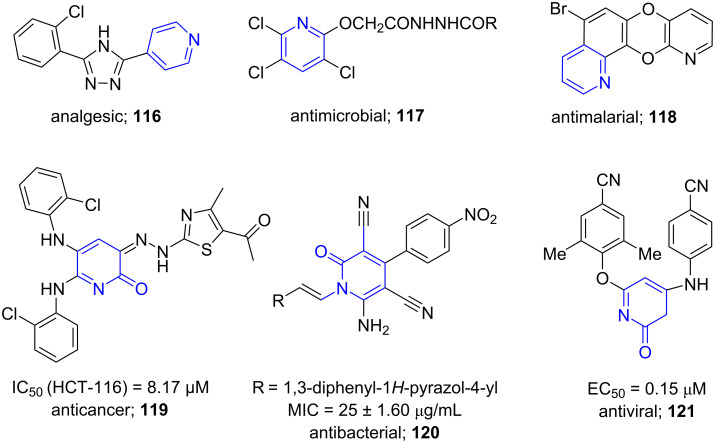
Biologically active molecules containing pyridine moiety.

Pyridines are six-membered ring systems consisting of five carbon atoms and one nitrogen atom. Highly substituted pyridines are known to show various pharmacological activities and are also found in various pharmaceuticals and biologically active molecules (**116**–**118**) [103]. Similarly, heterocycles having pyridone nucleus are pharmacologically important as they can act as potent anticancer (**119**), antibacterial (**120**) and antiviral agents (**121**, Figure 9) [104–106].

**Scheme 45:**
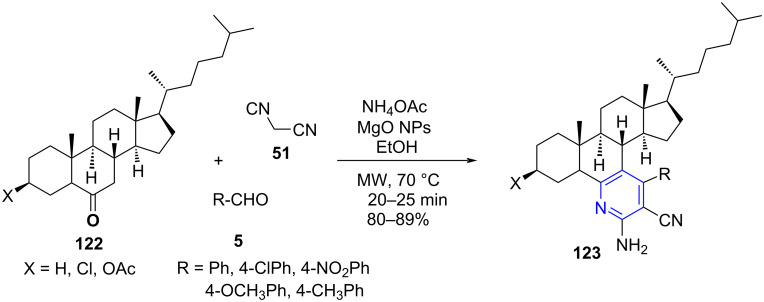
Synthesis of steroidal pyridines.

Shamsuzzaman and co-workers [107] demonstrated the microwave-assisted synthesis of steroidal pyridines **123** utilizing steroidal ketones **122**, aldehydes **5**, malononitrile (**51**)/methyl cyanoacetate and ammonium acetate as structural units and MgO nanoparticles as a catalyst in ethanol solvent. The reaction proceeded even in absence of a catalyst but resulted in a very low yield (Scheme 45). The authors methodically explored the surface defects of MgO such as edges, kinks and corners to advantage as they are regarded to enhance the efficiency of the catalyst by playing a crucial role in splitting the chemical bonds of the absorbed molecules [108].

**Scheme 46:**
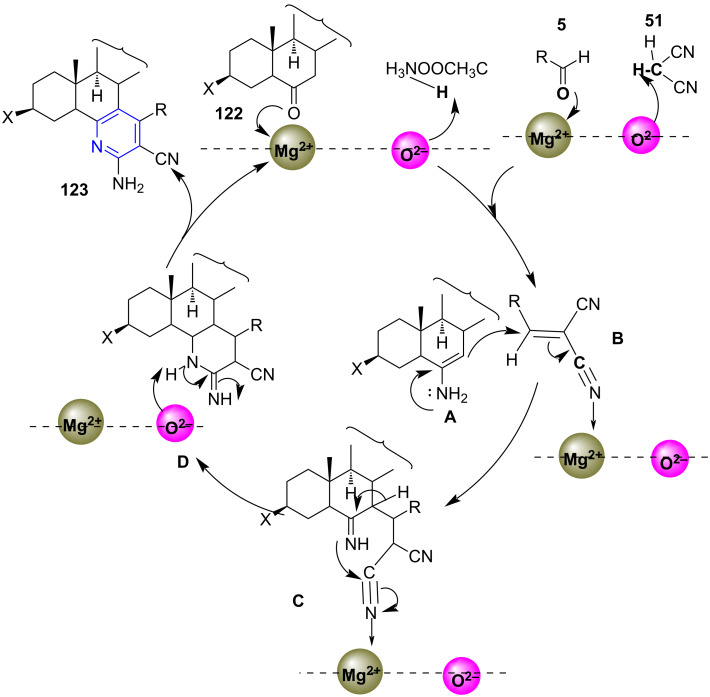
Proposed mechanism for steroidal pyridine.

The reusability of the heterogeneous catalyst is also an advantage of the stated strategy. The higher yields >82% obtained from the microwave-assisted protocol reveal its competency over the conventional method (79%) along with the time parameter wherein the time was reduced from hours to just minutes (6 h to 20 min). The protocol adroitly represents the efficiency of microwave and multicomponent strategy in the generation of complex molecules like steroids. The mechanism follows a pathway where an imine **A** is generated from the reaction between steroidal ketone and ammonium acetate. Simultaneously, the aldehyde and malononitriles undergoes Knoevenagel condensation resulting in alrylidene intermediate **B**. This is followed by Michael addition of imine **A** on the activated alrylidene intermediate **B** and subsequent intramolecular cyclization **C** and aromatization **D** affords the target molecules **123** (Scheme 46).

**Scheme 47:**
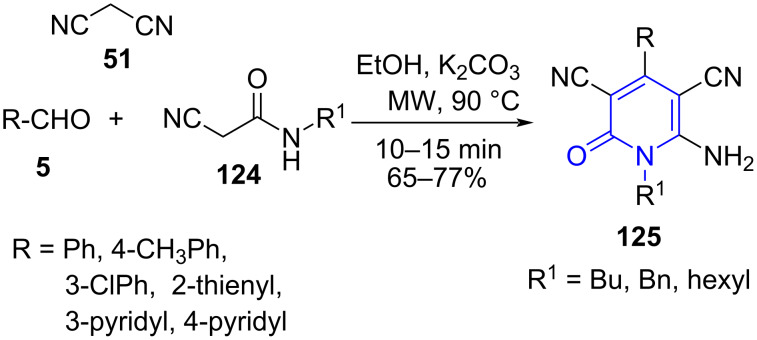
Synthesis of *N*-alkylated 2-pyridones.

*N*-alkylated pyridones are valuable scaffolds offering biological activity such as immunomodulators, memory-enhancers and anticancer agents [109–110]. A direct approach to achieve *N*-alkylated pyridones are less explored and those available present limitations such as poor selectivity and yields, expensive catalyst and poor chemoselectivityy [111–112]. Therefore, in search of a straightforward approach to such molecules. Mekheimer and co-workers [113] developed a protocol for the synthesis of *N*-alkylated 2-pyridones **125** utilizing a microwave-assisted three-component reaction of aldehydes **5**, malononitrile (**51**) and *N*-alkyl-2-cyanoacetamides **124** as structural units and K_2_CO_3_ as base promoter using EtOH as solvent (Scheme 47). The introduction of microwave drastically improved the yield from 65–77% to 81–94% along with reduction in time from 180 min to 15 min when a comparative study with conventional approach was performed.

**Scheme 48:**
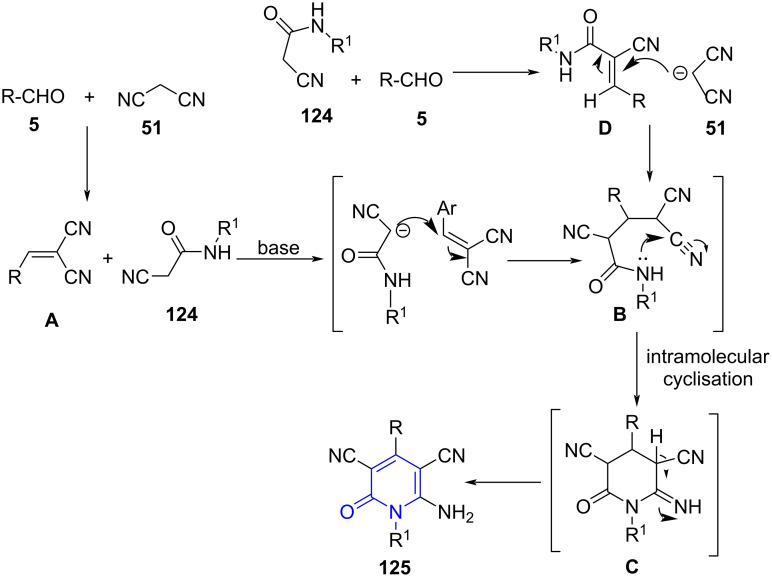
Two possible mechanisms for pyridone synthesis.

Two possible mechanisms have been postulated for the formation of **125**. The reaction is initiated by the Knoevenagel condensation of aldehyde and malononitrile (**51**) forming **A**. Further, a base-catalyzed Michael addition of acetamide over intermediate **A** results in the formation of adduct **B**. Adduct **B** then undergoes in situ cyclization through an intramolecular addition of nitrogen on amide which acts as a nucleophile to the nitrile and give intermediate **C**. The tautomerization of the imino to an amino group and subsequent auto-oxidation followed by aromatization affords the required products **125**. An alternative mechanism suggests the first Knoevenagel condensation between aldehyde and acetamide resulting in intermediate **D** to undergo Michael addition with malononitrile. The remaining pathway is similar as shown in Scheme 48.

**Scheme 49:**
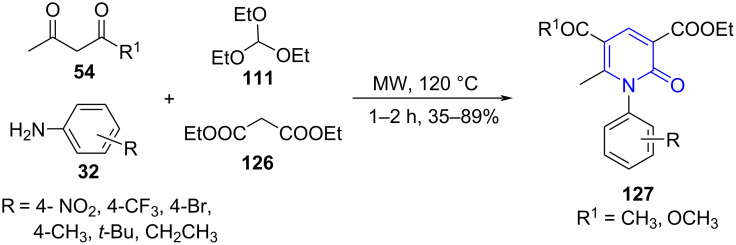
Synthesis of pyridone derivatives.

In the same direction, Huang and co-workers [114] for the first time reported a microwave-assisted four-component domino reaction involving acyclic 1,3-diketones **54**, amines **32,** diethyl malonate (**126**) and triethyl orthoformate (**111**) for the synthesis of substituted pyridone derivatives **127** at 120 °C under catalyst- and solvent-free conditions. The reaction proved adaptable even for uncommon amines such as simple alkylamines in good to moderate yields (Scheme 49). The initial assessment under refluxing conditions in presence of catalyst and solvent afforded the products in low yields (20–40%) in 2–3 h. Whereas catalyst- and solvent-free conditions under microwave irradiation spiked the yield to 84% in 30 min demonstrating the effectiveness of the technological approach.

**Scheme 50:**
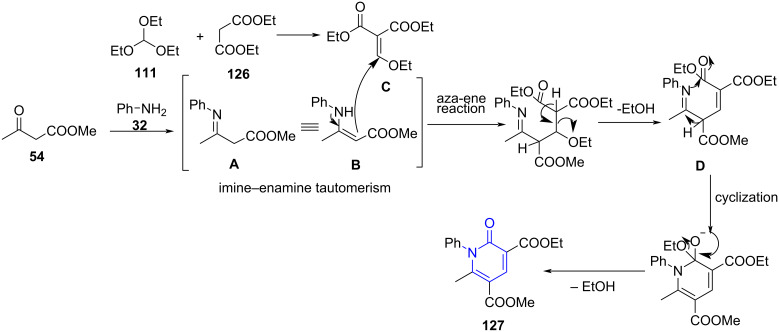
Postulated mechanism for synthesis of pyridone.

A plausible mechanism including a self-sorting system was suggested. A simple condensation of acyclic 1,3-diketone and amine results in intermediate **A** formation. The intermediate **A** undergoes imine–enamine tautomerization and affords intermediate enaminone **B**. The reaction of intermediate **B** with diethyl ethoxymethylenemalonate **C** forms intermediate **D** via an aza-ene mechanism with the loss of one molecular EtOH. A consecutive intramolecular cyclization of **D** with loss of ethanol ultimately yields the desired products **127** (Scheme 50).

#### 8.2 Fused pyridines

**Figure 10:**
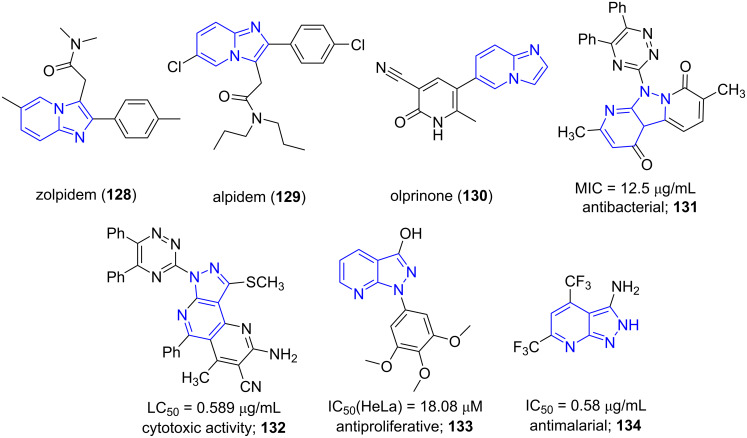
Biologically active fused pyridines.

Fused pyridines have been profoundly known for various pharmacological activities. Moreover, the imidazo[1,2-*a*]pyridine core is found in various drugs like zolpidem (**128**), alpidem (**129**), olprinone (**130**). They are also promising antiviral, antiulcer, anxiolytic, antiherpes agents [115–119]. Similarly, pyrazolo-pyridines are found to be potent antibacterial (**131**), cytotoxic (**132**), antiproliferative (**133**) and antimalarial (**134**) agents (Figure 10) [120–121].

**Scheme 51:**
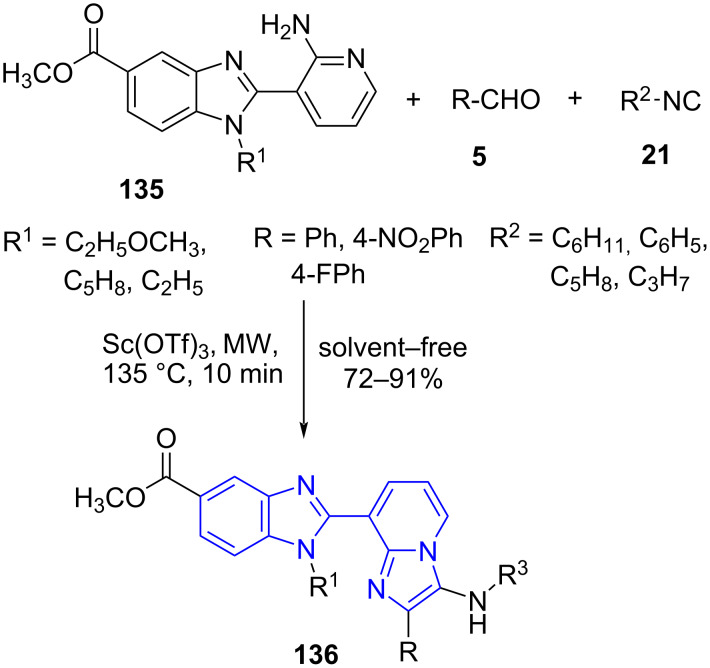
Benzimidazole-imidazo[1,2-*a*]pyridines synthesis.

**8.2.1 Imidazo[1,2-*a*]pyridine:** Sun and co-workers [122] accomplished the first systemic synthesis of biologically interesting bisheterocycles. A three-component microwave-assisted reaction of substituted benzimidazole-linked aminopyridine **135**, aldehydes **5** and isocyanide **21** using Sc(OTf)_3_ as the catalyst under solvent-free conditions resulted in substituted benzimidazole-imidazo[1,2-a]pyridines **136** (Scheme 51). The method developed employs a Groebke−Blackburn−Bienaymé reaction as the key transformation and facilitates the integration of two pharmacophores in one framework further enhancing the applicability in drug discovery with increased chemical space. The efficiency of the microwave technology was effectively explored by the authors in the synthesis of benzimidazole-linked aminopyridine, wherein every step for the construction of **135** was aided in the presence of microwave.

**Scheme 52:**
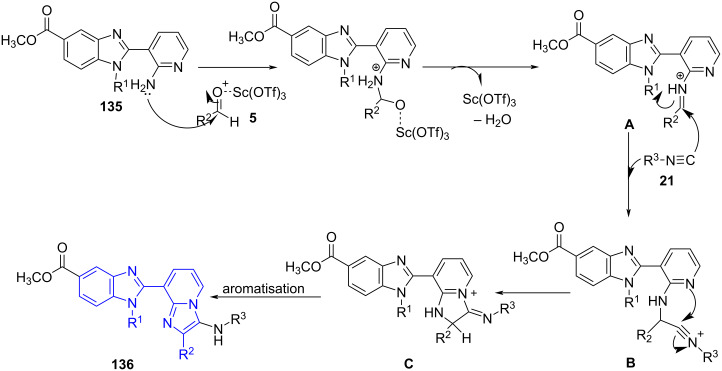
Mechanism for the synthesis of benzimidazole-imidazo[1,2-*a*]pyridines.

The possible mechanistic investigation indicates the formation of imine **A** by condensation between benzimidazole-linked aminopyridine and Lewis acid activated aldehyde which further on nucleophilic addition with substituted isocyanide leads to intermediate **B**. A 5-*exo*-*dig* intramolecular cyclization with isocyanide aids in the formation of the imidazo[1,2-*a*]pyridine intermediate **C**. The final product benzimidazoloimidazo[1,2-*a*]pyridine **136** was obtained by rearomatization of the intermediate **C** (Scheme 52).

**Scheme 53:**
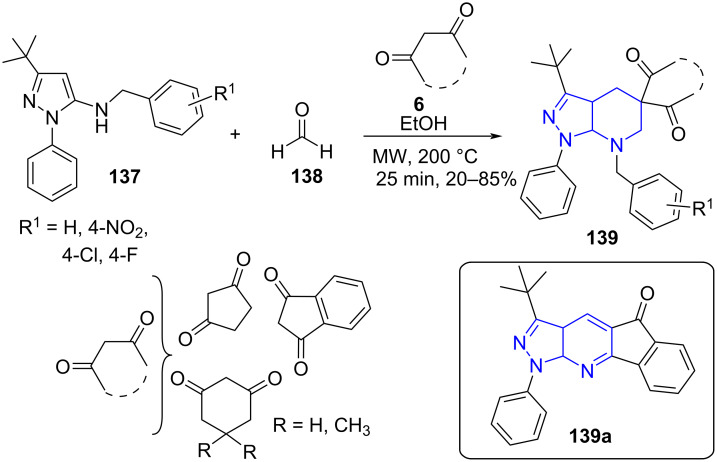
Synthesis of pyrazolo[3,4-*b*]pyridine-5-spirocycloalkanedione derivatives.

**8.2.2 Pyrazolopyridine:** Quiroga and co-workers [123] envisioned an environmentally benign three-component microwave-assisted synthesis of pyrazolo[3,4-*b*]pyridine-5-spirocycloalkanedione **139** derivatives via a reaction between 5-(4-R-benzyl amino)pyrazoles **137**, cyclic 1,3-diketones **6** and formaldehyde (**138**) in ethanol as solvent. The protocol shows good functional group tolerance with both EDG and EWG on pyrazoles resulting in moderate to good yields (Scheme 53). An interesting observation revealed that employing indanedione as the cyclic diketone directed the formation of an aromatized molecule **139a** instead of the expected spiro product. The authors rationalized **139a** formation through a competitive intramolecular cyclo condensation over intermolecular cyclo condensation reaction with loss of benzyl alcohol delivering a stable aromatized product. Although the yields were comparable under reflux and microwave approach, the conventional approach provided access to the desired molecule in 24 h whereas the microwave assistance exponentially reduced the reaction time to 25 min.

**Scheme 54:**
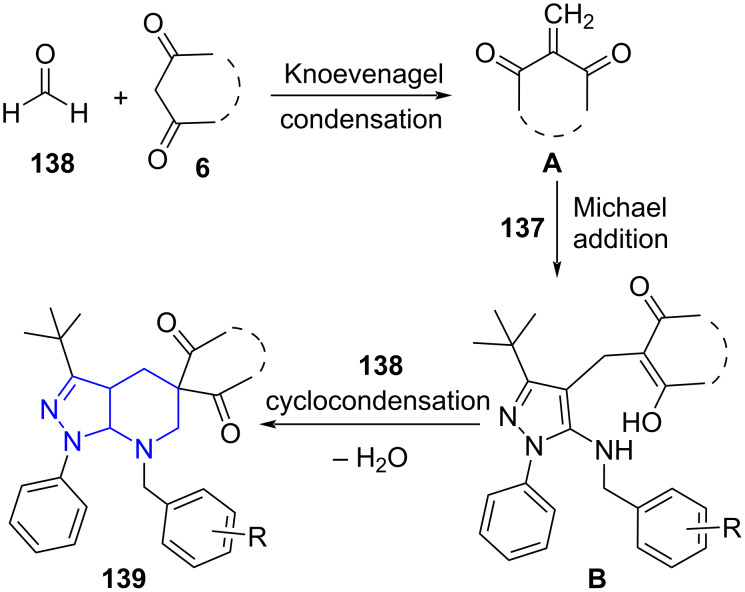
Proposed mechanism for spiro-pyridines.

A possible mechanism for the condensation reveals a straight Knoevenagel condensation between **138** and cyclic 1,3-diketones directing to Knoevenagel adduct **A**. A Michael type addition at the C-4 position of **137** with adduct **A** results in intermediate **B**. Finally in presence of another molecule of formaldehyde intermediate **B** undergoes cyclocondensation to yield the spiro product **139**, whereas for **139a** intermediate **B** falls prey to intramolecular cyclo condensation (Scheme 54).

**Scheme 55:**
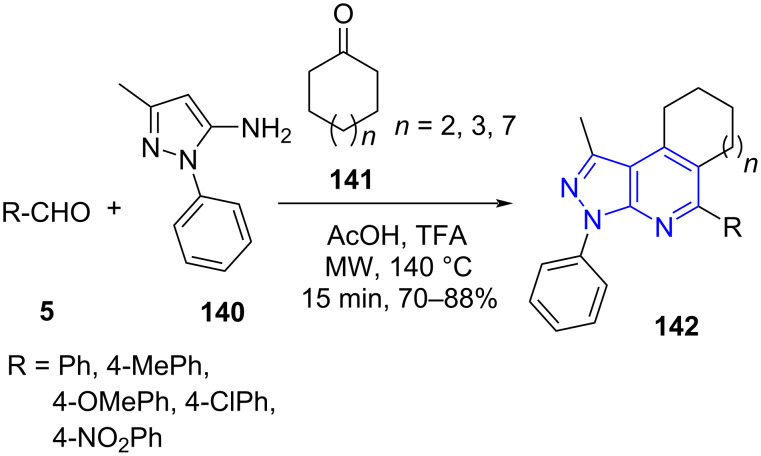
Functionalized macrocyclane-fused pyrazolo[3,4-*b*]pyridine derivatives.

The synthesis of regioselectively functionalized macrocyclane-fused pyrazolo[3,4-*b*]pyridine derivatives **142** was demonstrated by Jiang and co-workers [124] by employing aldehydes **5**, 5-methyl-3-aminopyrazole (**140**) and cycloketones **141** as building blocks in a one-pot manner with AcOH and TFA as promoter under microwave irradiation. This method stands out with its high efficiency and shorter reaction time to produce the macrocyclane-fused pyrazolo[3,4-*b*]pyridine skeleton (Scheme 55). The above protocol offers regioselectively 2-arylated pyrazolopyridines which the other reported protocols failed to produce with similar starting material. The previous reports produced 4-arylpyrazolopyridines [125–127].

**Scheme 56:**
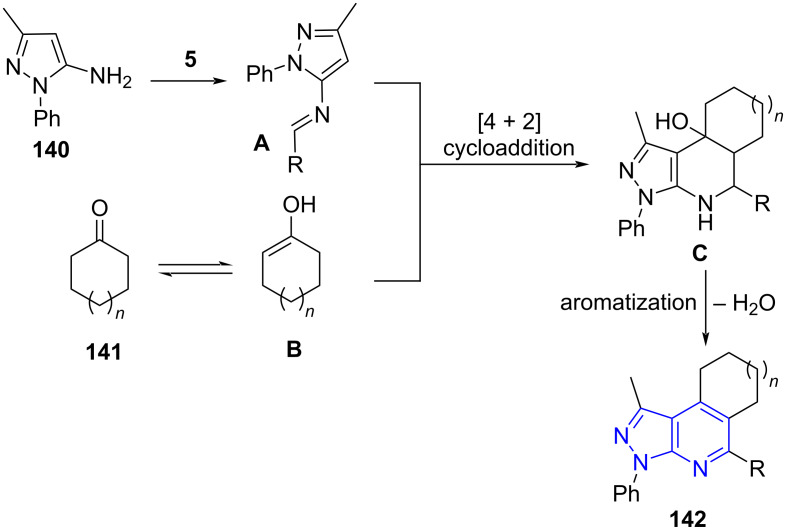
Mechanism postulated for macrocyclane-fused pyrazolo[3,4-*b*]pyridine.

The authors postulated the mechanism as depicted in Scheme 56 wherein cycloketone **141** in presence of AcOH exists in equilibrium with enol form **B**. The imine intermediate **A** (condensation of aldehyde and pyrazolylamine) surrenders to a [4 + 2] cycloaddition with the enol form **B** and result in cycloaddition adduct **C**. A further dehydration with concomitant aromatization yields the desired products **142**.

**Scheme 57:**
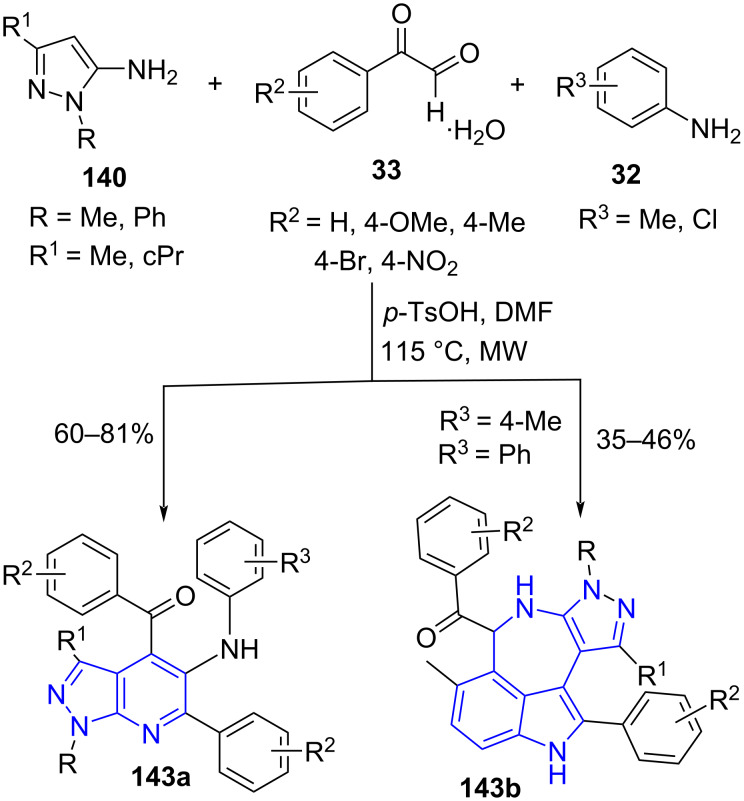
Generation of pyrazolo[3,4-*b*]pyridines.

A 6π-electrocyclization reaction was efficiently explored by Jiang and co-workers [128] for the construction of pyrazolo[3,4-*b*]pyridines **143a**. This approach consists of four-component reactions involving substituted pyrazolylamine **140**, two molecules of arylglyoxal **33** and amine **32** under microwave irradiation with *p*-TsOH as a catalyst for the generation of a library of pyrazolopyridines in good yields (Scheme 57). The methyl substitution at the C-4 position of the aniline (*p*-toluidine-**32b**) led to the formation of azepino[5,4,3-*cd*]indole products **143b**.

**Scheme 58:**
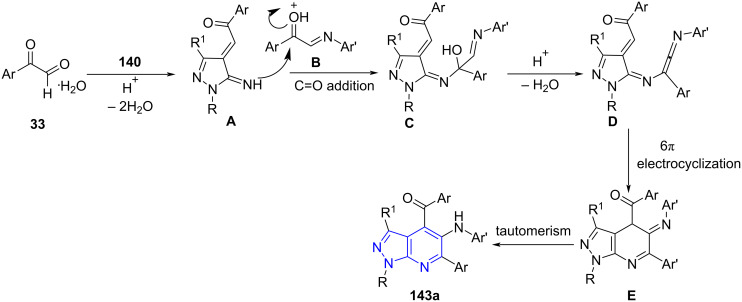
Proposed mechanism for the synthesis of pyrazolo[3,4-*b*]pyridines.

The proposed mechanism in Scheme 58 depicts the formation of intermediate **A** from condensation of arylglyoxal and pyrazolylamine protonated by *p*-TsOH with subsequent dehydration. An enone intermediate generated from the carbonyl addition reaction of intermediate **A** with imine **B** transforms into the allene intermediate **D**. Finally, an intramolecular 6π-electrocyclization and tautomerism results in the desired products **143a**.

**Scheme 59:**
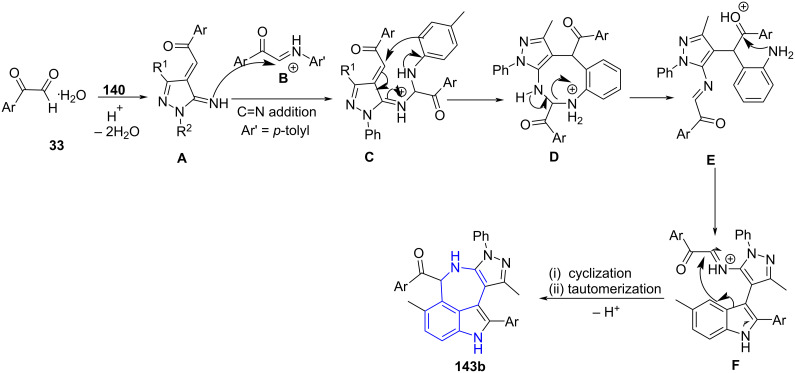
Proposed mechanism for the synthesis of azepinoindole.

The authors proposed a mechanism for azepinoindoles (Scheme 59) [128] wherein acid-catalyzed protonation of arylglyoxal monohydrate followed by dehydration and addition of electron-rich pyrazolylamine led to the formation of intermediate **A**. Simultaneously, intermediate **B** (condensation of arylgyoxal and primary amine **32b** undergoes C=N addition with intermediate **A**. Intramolecular cyclization of **C** yields a macrocyclic intermediate **D** which is followed by *p*-TsOH-promoted ring opening resulting in imine intermediate **E**. A consecutive intramolecular cyclization and tautomerization yields azepino[5,4,3-*cd*]indoles **143b**.

### 9 Quinolines

**Figure 11:**
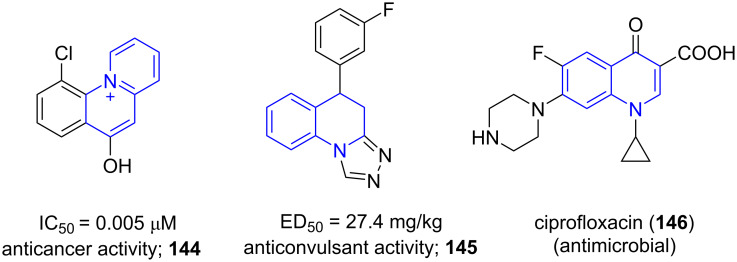
Pharmaceutically important molecules with quinoline moiety.

Quinolines are bicyclic aromatic heterocycles consisting of a fused pyridine and benzene ring. Quinoline and its derivatives are important both from synthetic as well as biological perspective owing to their plethora of pharmacological activities. They are potent anticancer (**144**), antimicrobial (**145**), and anticonvulsant agents (**146**, Figure 11) [129].

**Scheme 60:**
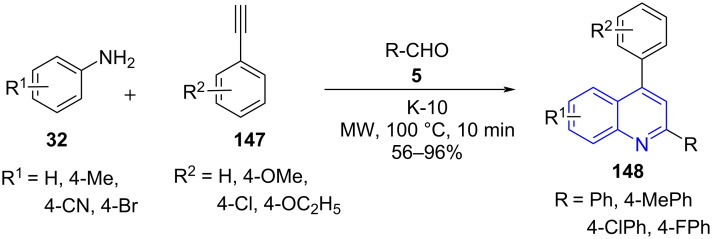
Povarov-mediated quinoline synthesis.

Their occurrence in various natural products, pharmaceuticals and materials science [130–131] inspired Török and co-worker [132] to design an efficient synthetic approach involving a multicomponent reaction for the synthesis of 2,4-diarylquinolines **148**. Substituted amines **32**, aldehydes **5** and alkynes **147** were used as substrates by utilizing solid acid catalyst K-10 under microwave irradiation and solvent-free conditions (Scheme 60). The conventional heating under similar conditions afforded the target molecule in low yield (40%) with an extended reaction time of 3 h which clearly suggests the effectiveness of the microwave approach delivering >56% yield in 30 min of reaction period.

**Scheme 61:**
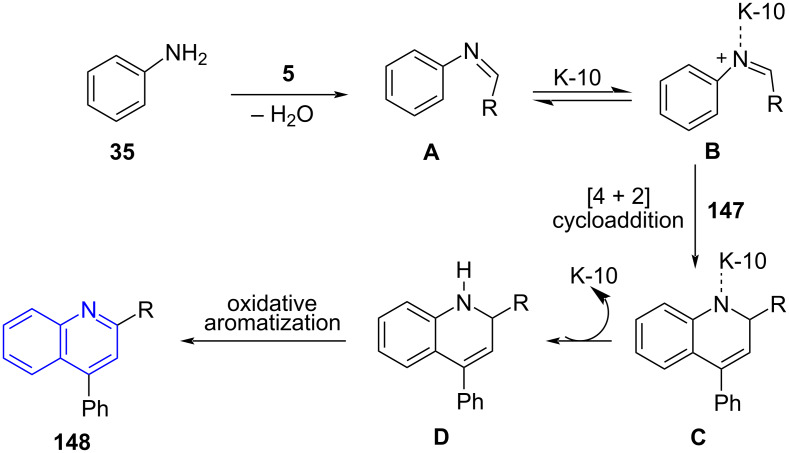
Proposed mechanism for Povarov reaction.

The stability and the recyclability of the solid catalyst were maintained efficiently up to five cycles with excellent yields (92–96%). The formation of compound **148** proceeds by the condensation of amine and aldehyde to give rise to an aldimine intermediate **A** followed by a Povarov-type multicomponent reaction involving a [4 + 2] cycloaddition of the alkyne with K-10 activated imine **B** resulting in intermediate **C**. The dihydroquinoline intermediate **D** undergoes oxidative aromatization to afford the final product (**148**, Scheme 61).

**Scheme 62:**
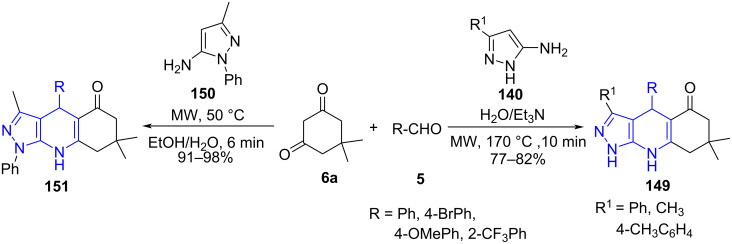
Synthesis of pyrazoloquinoline.

In 2011, Chebanov and co-workers [133] reported an aqueous medium base-catalyzed three-component reaction for the synthesis of substituted pyrazolo[3,4-*b*]quinolin-5-ones **149** involving aldehydes **5**, cyclic 1,3-diketone such as dimedone (**6a**) and substituted 1*H*-pyrazol-5-amine **140** irradiated at 170 °C using microwaves (Scheme 62). The stated protocol reveals an efficient strategy to produce fused pyrazoloquinolines in good to excellent yields with good functional group tolerance. In 2019, a very similar approach was demonstrated by Jonnalagadda and co-workers [134] with 5-amino-3-methyl-1-phenylpyrazole (**150**), aldehyde **5**, cyclic 1,3-diketone like dimedone (**6a**) under microwave irradiation in a solvent mixture of water and ethanol at 50 °C. The constructed pyrazolo[3,4-*b*]quinoline derivatives **151** were delivered in excellent yield with good functional group tolerance (Scheme 62).

**Scheme 63:**
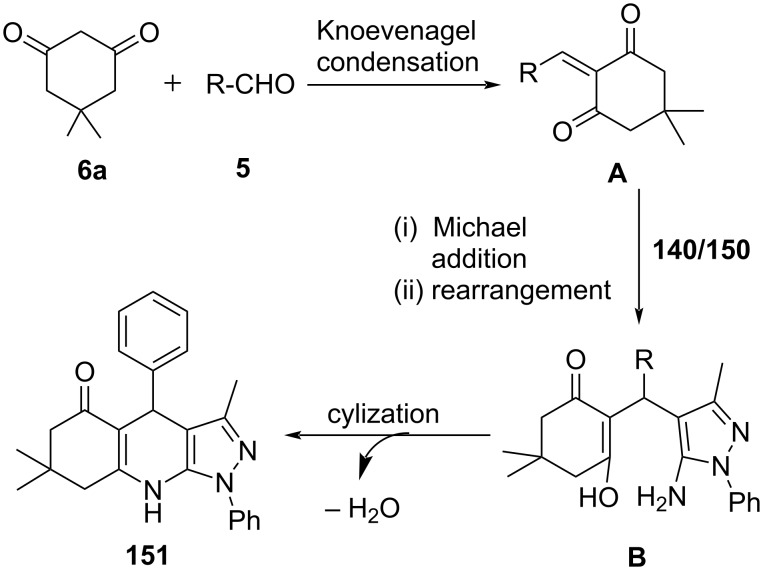
Plausible mechanism for pyrazoloquinoline synthesis.

The proposed mechanism in Scheme 63 revealed the formation of Knoevenagel intermediate **A** between aldehyde and dimedone. Michael addition of pyrazole **140** or **150** with intermediate **A** results in a rearranged adduct **B**, which then converts into the desired products **149** and **151** after subsequent cyclization and dehydration.

### 10 Quinazolinone

**Figure 12:**
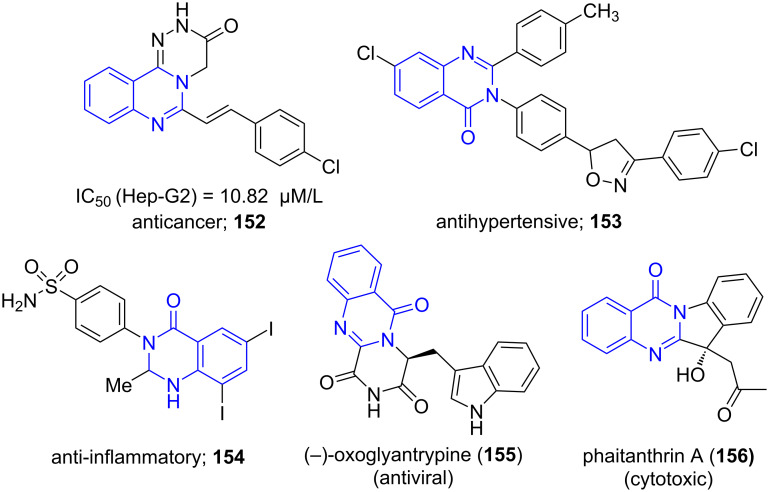
Quinazolinones as pharmacologically significant scaffolds.

Quinazoline is an organic heterobicyclic compound characterized by a naphthalene ring with the carbon atoms at positions 1 and 3 replaced by nitrogen atoms. Quinazolinones are heterocyclics represented as quinazoline ring with a keto group. Figure 12 depicts quinazolinones demonstrating various pharmacological properties like anticancer (**152**), antihypertensive (**153**) and anti-inflammatory (**154**) [135–136]. Many quinazolinone ring-containing alkaloids have been widely used in drug discovery and development protocols (**155** and **156**) [137].

#### 10.1 5,6-Dihydroquinazolinones

**Scheme 64:**
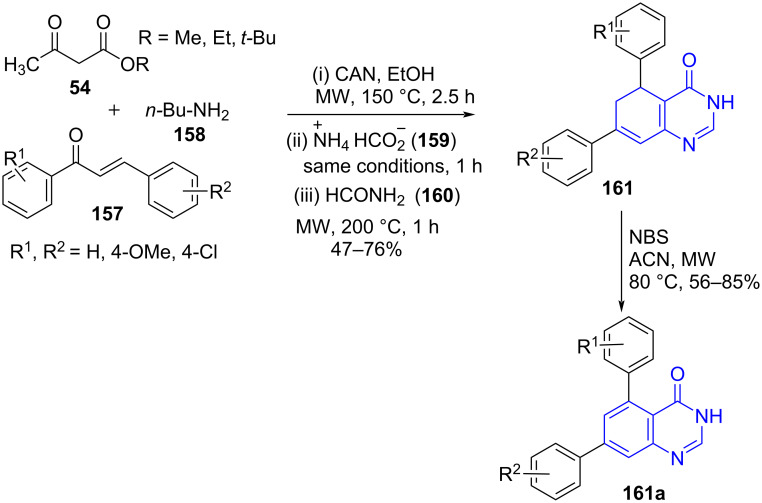
Four-component reaction for dihydroquinazolinone.

Menéndez and co-workers [138] described an efficient microwave-assisted sequential four-component reaction of chalcones **157**, acyclic 1,3-diketone **54**, butylamine (**158**) and ammonium formate (**159**) using CAN as a catalyst and ethanol as solvent. This is followed by sequential addition of formamide (**160**) under microwave irradiation to yield polysubstituted 5,6-dihydroquinazolinones **161** in good to moderate yields (Scheme 64). The protocol exemplifies the use of MW-assisted MCR for the construction of the aromatic ring from a simple aliphatic chain. Non-chromatographic techniques for purification of the products further added to the list of advantages to the method. The authors also succeeded in developing a metal free *N*-bromosuccinimide-mediated MW-assisted halogenation elimination sequence resulting in aromatization of dihydroquinazolinones **161a** reducing the use of traditional highly polluting dehydrating agents [139].

**Scheme 65:**
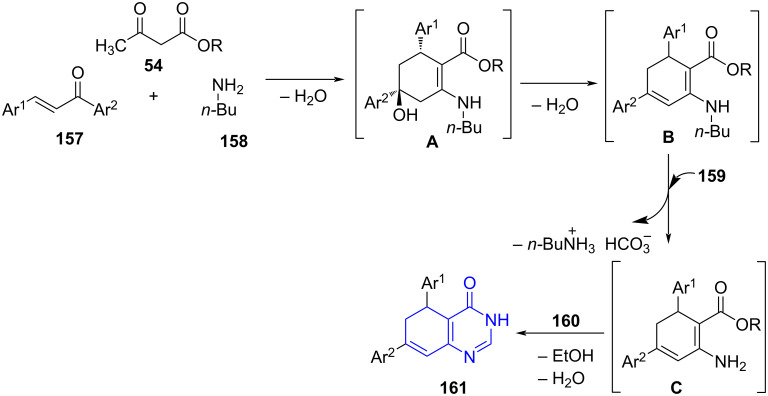
Proposed mechanism for dihydroquinazolinones.

The mechanism proposed in Scheme 65 illustrates the formation of intermediate **A** from a reaction between chalcones, acyclic 1,3-diketone and butylamine. The removal of a water molecule from intermediate **A** leads to the dehydrated intermediate **B** which upon reaction with ammonium formate under microwave irradiation results in intermediate **C**. The addition of formamide finally produces the desired polysubstituted 5,6-dihydroquinazolinones **161** with ethanol and water as byproducts.

**Scheme 66:**
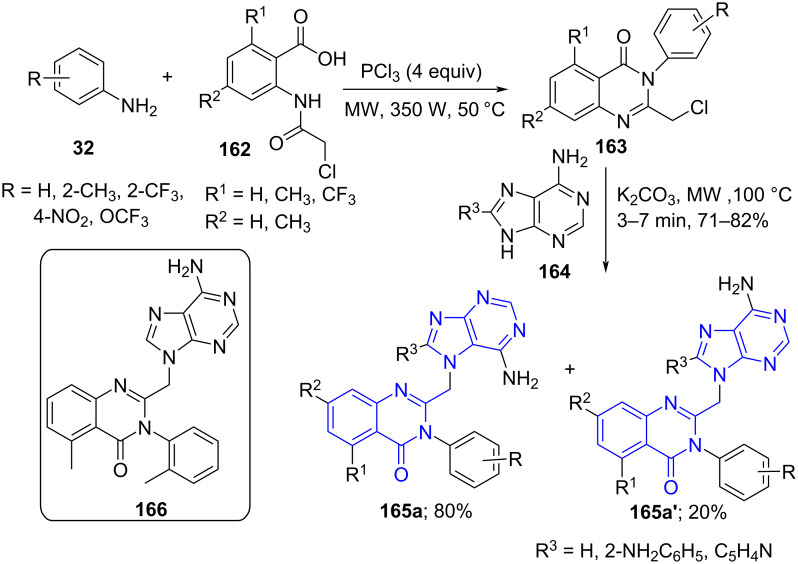
Synthesis purine quinazolinone and PI3K-δ inhibitor.

Similarly, Sawant and co-workers [140] demonstrated a one-pot multicomponent reaction under microwave irradiation to synthesize purine-based quinazolinone derivatives **165**. The reaction follows a sequential addition of amines **32** and aminobenzoic acids **162** to form **163** with PCl_3_ as a cyclising agent under microwave conditions. A sequential addition of adenine **164** in the presence of a K_2_CO_3_ yields regioisomers of substituted purine quinazolinone **165** in an 80:20 ratio (Scheme 66). The authors observed a variation in the ratio of regioisomer formation with a slight deviation in reaction conditions such as microwave power, reaction time or temperature. The set protocol was successfully employed for the synthesis of structural analogues of IC87114 (**166**), first isoform-selective PI3K-δ inhibitor used as an anticancer agent [141].

#### 10.2 Fused quinazolinones

**Scheme 67:**
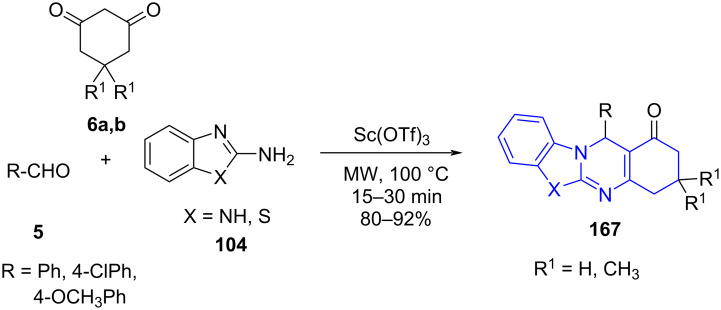
Synthesis of fused benzothiazolo/benzoimidazoloquinazolinones.

The fused substituted benzothiazolo/benzimidazoloquinazolinones **167** was achieved by Singh and co-workers [142] from aldehyde **5**, cyclic 1,3-diketones **6a**,**b** and 2-aminobenzoazoles **104** as the structural fragments with Sc(OTf)_3_ as catalyst under microwave irradiation in solvent-free conditions (Scheme 67). The catalytic activity of the catalyst was evident to remain intact even with three successive runs and provided with an environmentally benign and cost effective approach towards the construction of fused quinazolinones.

**Scheme 68:**
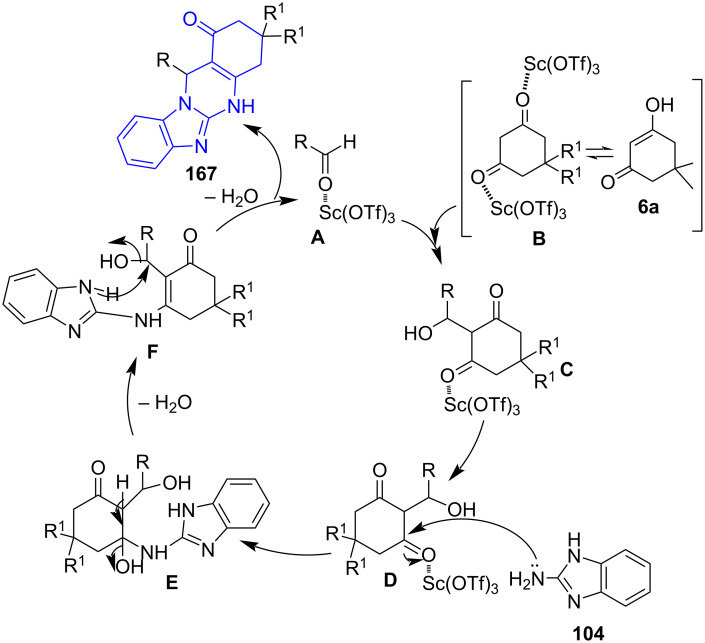
Proposed mechanism for fused benzothiazolo/benzoimidazoloquinazolinones.

Based on the literature [143–144], the authors deduced a plausible mechanism as described in Scheme 68. Initial activation of oxygen on the carbonyl group of cyclic 1,3-diketone **B** and aldehyde **A** by Sc(OTf)_3_ is followed by Knoevenagel condensation between these activated groups **C**. Sc(OTf)_3_ enhances the electrophilic character of oxygen by coordinating with carbonyl oxygen. This facilitated an easy attack on the carbonyl carbon **D** by the lone pair of nitrogen from 2-aminobenzazoles which stemmed the desired products **167** by dehydration (**E**) followed by intramolecular cyclization (**F**).

**Scheme 69:**
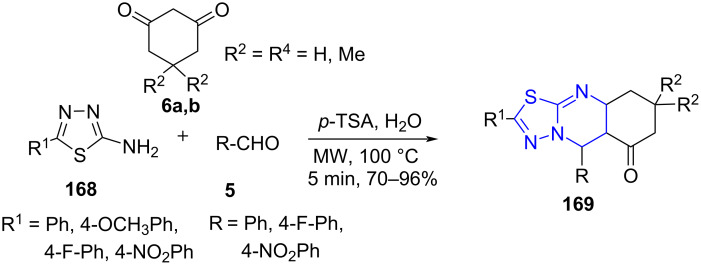
On-water reaction for synthesis of thiazoloquinazolinone.

The traditional methods for thiadiazoloquinazolinone synthesis possessed certain limitations, such as reduced yields, multi-step procedures and expensive starting materials [145–146]. On water chemistry has been in the scientific community for a while but has received little attention [147]. Focussing their efforts towards MWA-MCR on water, Sharma and co-workers [148] established a crafty construction of thiadiazolo[2,3-*b*]quinazolinones **169**. The on water reaction involved substituted 1,3,4-thiadiazol-2-amine **168**, aldehydes **5** and cyclic 1,3-diketones **6a,b** in an aqueous acidic medium of *p*-TSA (Scheme 69). A comparative study of conventional and microwave-assisted reactions clearly resulted in an exponential increase in yield from 78% to 96% and reduced reaction time from 6 h to 5 min with the microwave approach. Good functional group tolerance was demonstrated with all three reaction components.

**Scheme 70:**
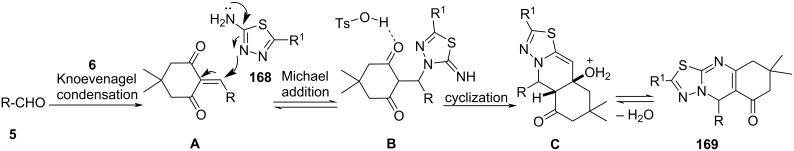
Proposed mechanism for the thiazoloquinazolinone synthesis.

A catalytic OH site present among one of the four water molecules at the interface of water and the organic layer is reasoned as the reaction center. Scheme 70 explains the initiation of the reaction with Knoevenagel condensation between aldehyde and cyclic diketone to form intermediate **A**. The water and *p*-TSA promoted Michael addition of intermediate **A** and thiadiazol-2-amine led to Michael adduct **B**. A subsequent cyclization **C** followed by dehydration produces thiadiazolo[2,3-*b*]quinazolinones **169**.

**Scheme 71:**
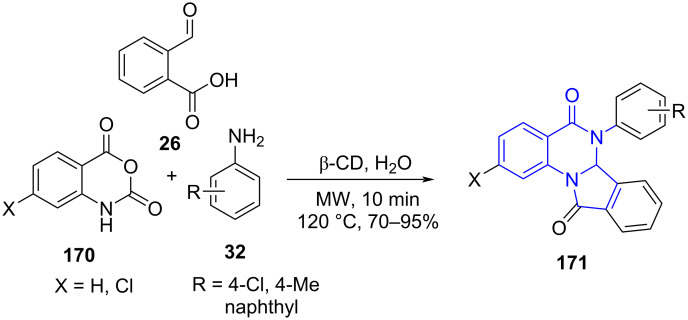
β-Cyclodextrin-mediated synthesis of indoloquinazolinediones.

In 2013, Pal and co-workers [149] reported a β-cyclodextrin-mediated synthesis of 6,6a-dihydroisoindolo[2,1-*a*]quinazoline-5,11-diones **171** in an aqueous medium. The strategy employed isatoic anhydride **170**, amines **32** and 2-formylbenzoic acid (**26**) as the building blocks under microwave irradiation (Scheme 71). The high selectivity of cyclodextrin is attributed to the hydrophobic cavities that facilitate the specific substrate binding and reactivity. The conventional method of reaction resulted in prolonged reaction time (14–16 h) and reduced yield whereas microwave assistance aided reduced time (10 min) with increased yield (up to 95%). The protocol provides a greener and faster approach towards such biologically effective motif's which under classical protocols are tedious to synthesize.

**Scheme 72:**
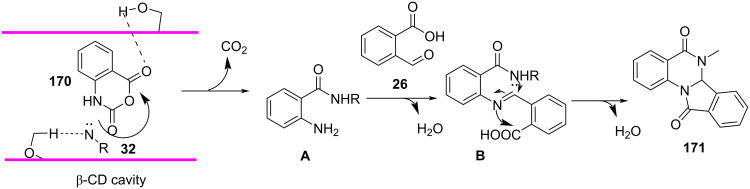
Proposed mechanism for synthesis of indoloquinazolinediones.

The plausible mechanism in Scheme 72 reveals the catalyst aided activation of anhydride carbonyl, followed by nucleophilic attack of amine results in a benzamide intermediate **A** generated in situ. A subsequent reaction of intermediate **A** with formylbenzoic acid directs imine intermediate **B** formation followed by a concurrent intramolecular cyclization involving the acid and amide groups generates the desired products **171**.

### 11 Triazoles

**Figure 13:**
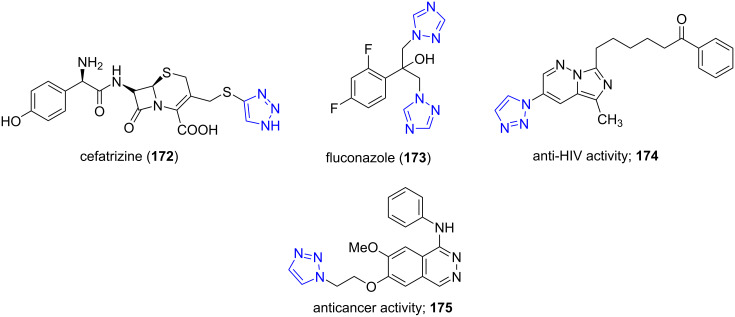
Triazoles-containing marketted drugs and pharmacologically active molecules.

1,2,3-Triazoles are heterocyclic compounds containing a five-membered ring with two carbons and three nitrogen atoms. They have emerged as core structural units in marketed drugs such as cefatrizine (antibiotic; **172**) and fluconazole (antifungal; **173**). Other medicinal activities explored are antiviral (**174**) and anticancer activity (**175**, Figure 13) [150–152].

**Scheme 73:**
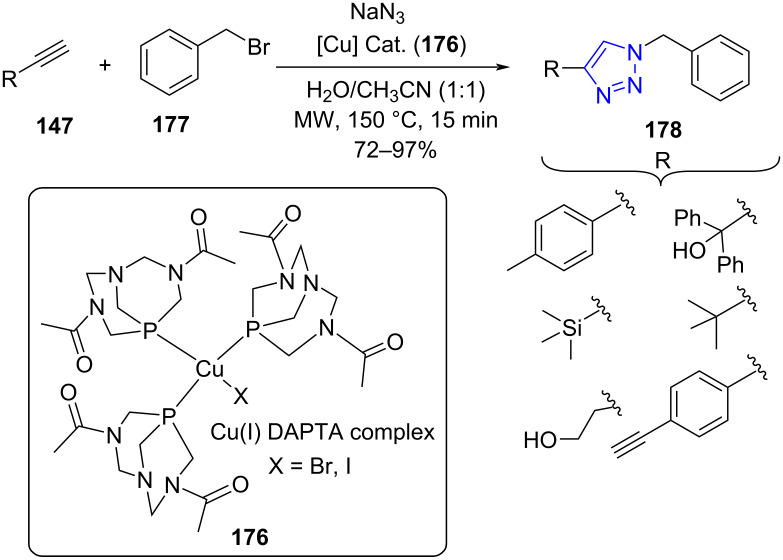
Cu(I) DAPTA-catalyzed 1,2,3-triazole formation.

Guedes da Silva and co-workers [153] successfully developed a protocol for the efficient synthesis of hydrosoluble, air stable Cu(I) DAPTA (3,7-diacetyl-1,3,7-triaza-5-phosphabicyclo[3.3.1]nonane, **176**) and further employed it as a catalyst in the Huisgen cycloaddition reaction for the synthesis of disubstituted 1,2,3-triazoles **178** using alkyne **147**, organic halide **177** and sodium azide in aqueous medium under microwave irradiation (Scheme 73). The reaction involves in situ-generated azide from organic halide which reacts with copper(I) acetylide to provide the corresponding 1,4-disubstituted 1,2,3-triazole. The cage-like DAPTA is a water soluble phosphine that can stabilize low oxidation state metals like copper hence used as ligands with copper to form complexes that catalyze the reaction. The catalyst can be recycled and showed good reactivity up to two cycles with good yields. The conventional heating technique successfully generated the desired molecules in 14 h whereas the microwave-assisted method considerably reduced the time to 15 min with similar yields.

**Scheme 74:**
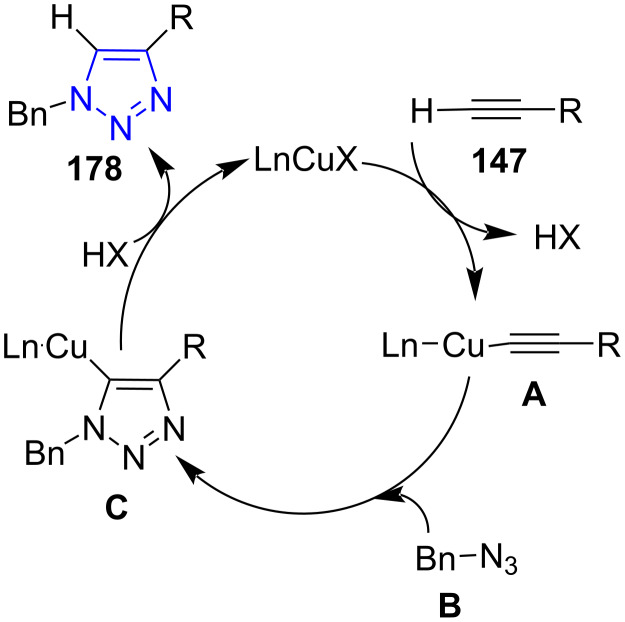
Mechanism for Cu(I) DAPTA-catalyzed triazole formation.

The possible mechanism of the reaction employing copper as catalyst proceeded with the formation of Cu acetylide complex **A** through the coordination of alkyne to Cu(I) which further reacts with benzyl azide **B** (formed from **177** and azide) leads to the formation of an intermediate **C**. This triazolide intermediate undergoes protonation to afford the final product, i.e., 1,4-disubstituted 1,2,3-triazoles **178**, thereby completing the catalytic cycle (Scheme 74).

**Scheme 75:**
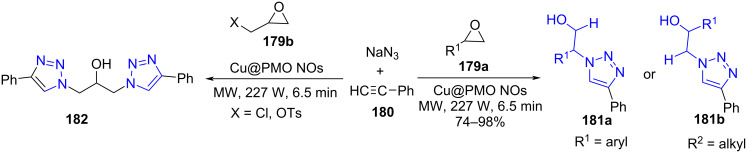
Synthesis of β-hydroxy-1,2,3-triazole.

Naeimi and co-workers [154] introduced a copper-imprinted periodic mesoporous organosilica nanocomposite (Cu@PMO NC), a catalyst employed for the synthesis of β-hydroxy-1,2,3-triazoles **181** in an aqueous medium. The strategy engaged epoxides **179** and sodium azide for the in situ generation of organo azides entrapped by the catalyst for further reaction with acetylide **180** under microwave irradiation. Under similar conditions, epoxides with a good leaving group direct the formation of bistriazoles **182** (Scheme 75). The method proposes a number of advantages, such as environmentally benign conditions like a solvent (water), energy consumption (microwave), reduced time (6–7 min), good to excellent yields (74–95%) and recyclability and reusability of the catalyst. The observation suggested the efficiency of the catalyst remains intact even after six cycles.

**Scheme 76:**
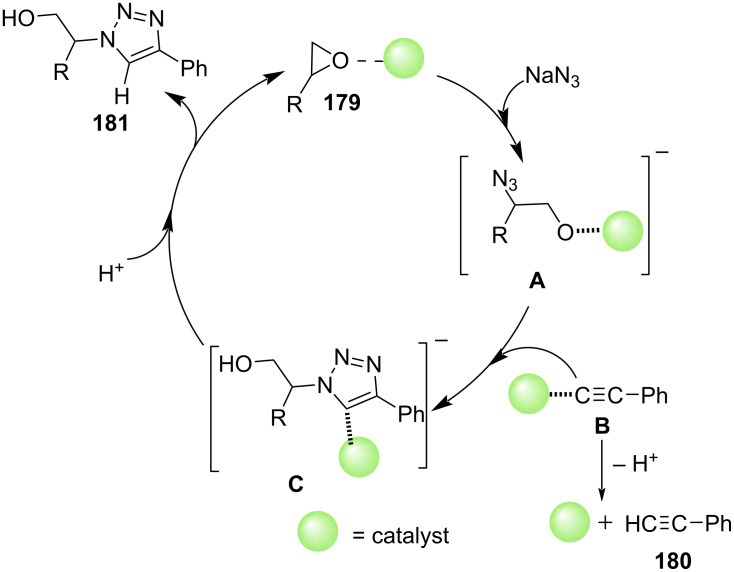
Proposed mechanism for synthesis of β-hydroxy-1,2,3-triazoles.

A plausible mechanism explained in Scheme 76 depicts the catalyst-mediated epoxide ring-opening by azide forms azido-aryl ethanol intermediate **A**. Cu(II) acetylide complex **B** undergoes the classical 1,3-dipolar cycloaddition product Cu(II) β-hydroxytriazolide (**C**). The protonlysis of the complex **C** directs the formation of the final desired product of β-hydroxytriazolide **181**.

**Scheme 77:**
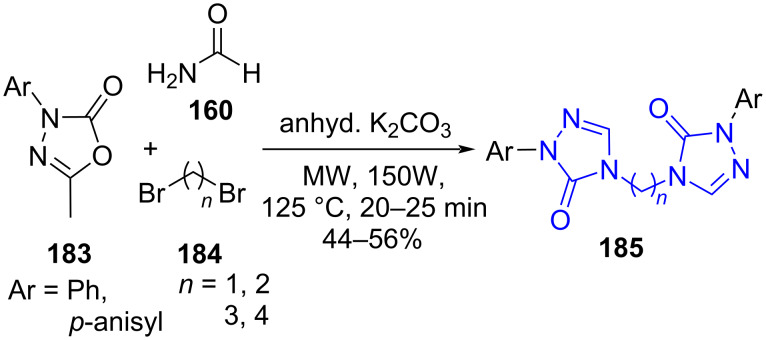
Synthesis of *bis*-1,2,4-triazoles.

1,2,4-Triazoles have carved a niche as potent antifungal agents with fluconazole (**173**) as the representative drug of this category. The bistriazoles inspired skeletons constructed by Kamble and co-workers [155] demonstrated the efficient synergistic application of microwave and multicomponent reactions. Two strategies were studied to optimize the reaction yield. The one-pot reaction involving 1,3,4-oxadiazol-2(3*H*)-one **183**, formamide (**160**) and dibromoalkanes **184** in presence of K_2_CO_3_ under solvent-free conditions aided the target bistriazoles **185** in moderate yield (Scheme 77).

**Scheme 78:**
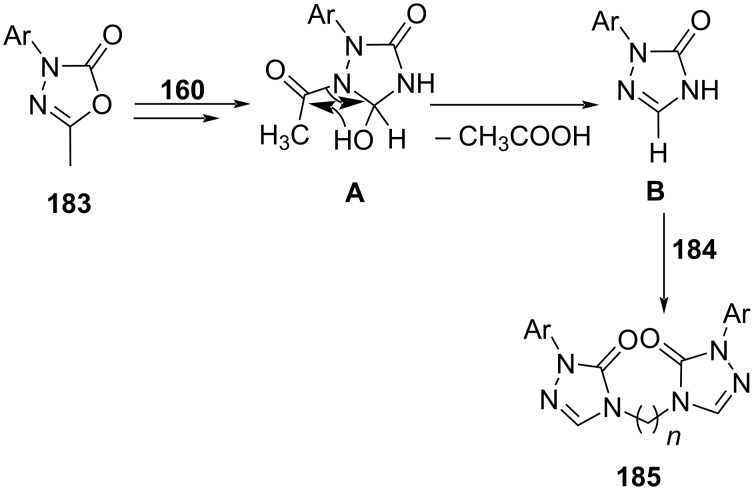
Proposed mechanism for *bis*-1,2,4-triazoles synthesis.

An alternative approach suggested the sequential addition of 1,3,4-oxadiazol-2(3*H*)-one **183** and formamide (**160**) followed by dibromoalkanes **184** under similar conditions resulted in higher yields (72–93%) than the one-pot approach. The authors also observed that the multicomponent strategy under the conventional method at 200 °C failed to produce the desired molecule whereas the sequential addition under the traditional refluxing method resulted in the product in moderate yield. Such an observation clearly establishes the dominance of MWA-MCR in the generation of valuable pharmacophores. The synthesized molecules studied for antifungal activities showed moderate to excellent activity against *A. niger*, *A. flavus*, *T. atroviridae*, *P. chrysogenum*, and *C. albicans.* Scheme 78 depicts the ring insertion of nitrogen into 1,3,4-oxadiazol-2(3*H*)-one directed by formamide followed by demethylation at C-5. Two consecutive nucleophilic substitutions with dibromoalkanes yields bistriazoles **185**, the target molecule.

### 12 Miscellaneous

#### 12.1 Thiazoles

**Figure 14:**
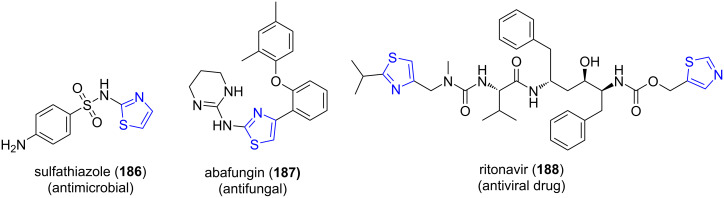
Thiazole containing drugs.

**Scheme 79:**
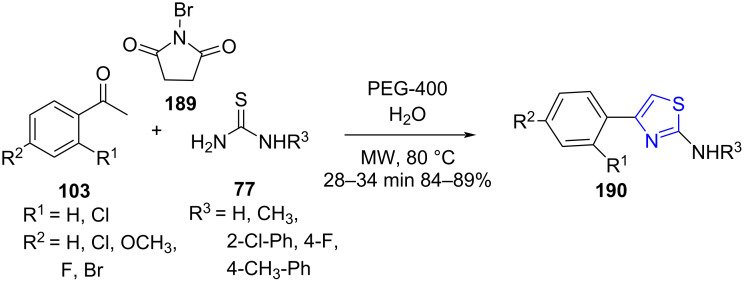
Synthesis of a substituted thiazole ring.

Thiazoles are employed in many medicinally important drugs (Figure 14) such as sulfathiazole (**186**), abafungin (**187**) and ritonavir (**188**) [156]. In 2017, Wagare and co-workers [157] demonstrated the construction of substituted thiazole ring **190** by utilizing a three-component reaction involving NBS (**189**), aromatic ketones **103** and thioureas **77** under microwave radiation using PEG-400 and water as a medium at 80–85 °C (Scheme 79).

**Scheme 80:**
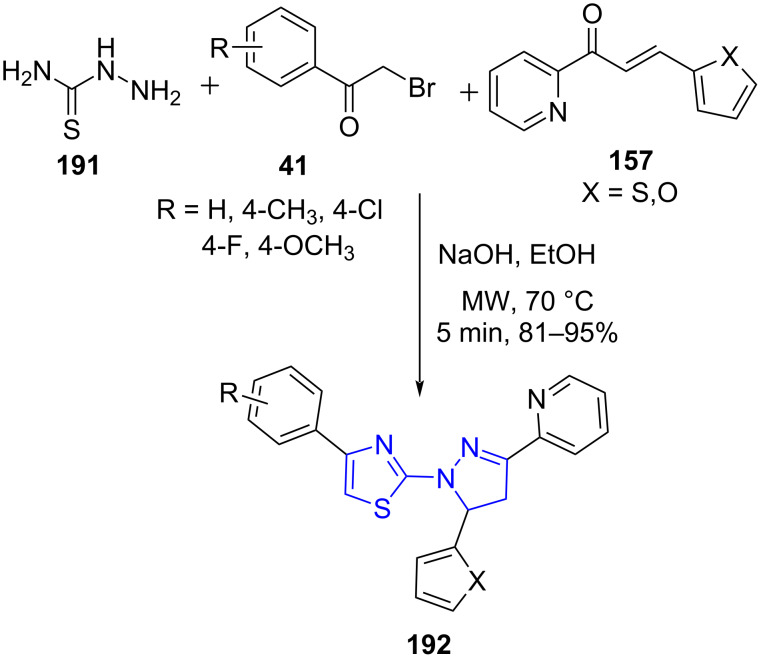
Synthesis of pyrazolothiazoles.

Recently, Vedula and co-workers [158] designed a facile and efficient base-catalyzed microwave-assisted three-component reaction between thiosemicarbazide (**191**), substituted chalcones **157** with substituted phenacyl bromides **41** in EtOH for the construction of pyrazolothiazoles **192**. A comparative study of the conventional refluxing method with the microwave-assisted protocol depicted the efficiency of microwave technology in increasing the yield from 82% to 95% in reduced time from 4 h to 5 min. The library of molecules so generated was found to be active against different cancer cell lines which make this protocol useful for the generation of molecules that can act as lead for pharmacologically active moieties (Scheme 80). The reaction is believed to proceed via the synthesis of a Hantzsch thiazole followed by condensation with substituted chalcones.

#### 12.2 Synthesis of chromenes

**Figure 15:**
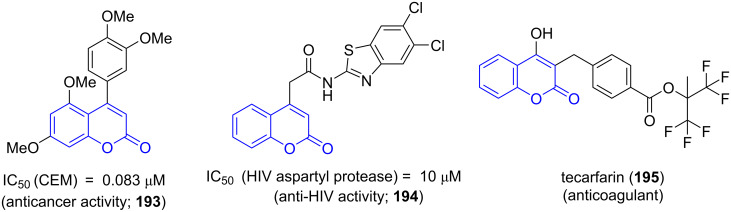
Chromene containing drugs.

Chromene and their derivatives are attributed to exhibit various biological activities (Figure 15) such as anticancer (**193**), antiviral (**194**) and anticoagulant like tecarfarin (**195**) [159].

**Scheme 81:**
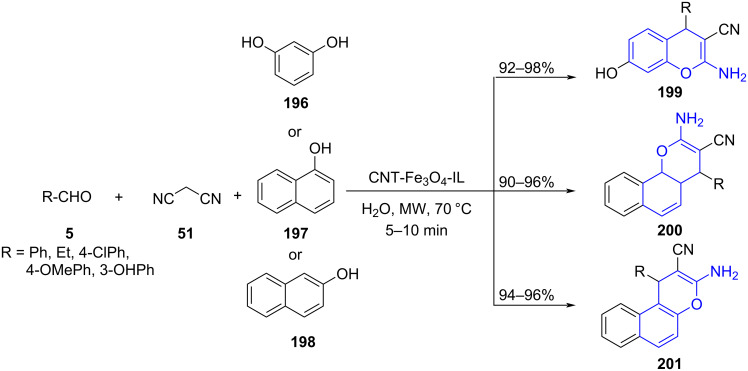
Magnetic nanocatalyst-mediated aminochromene synthesis.

**Scheme 82:**
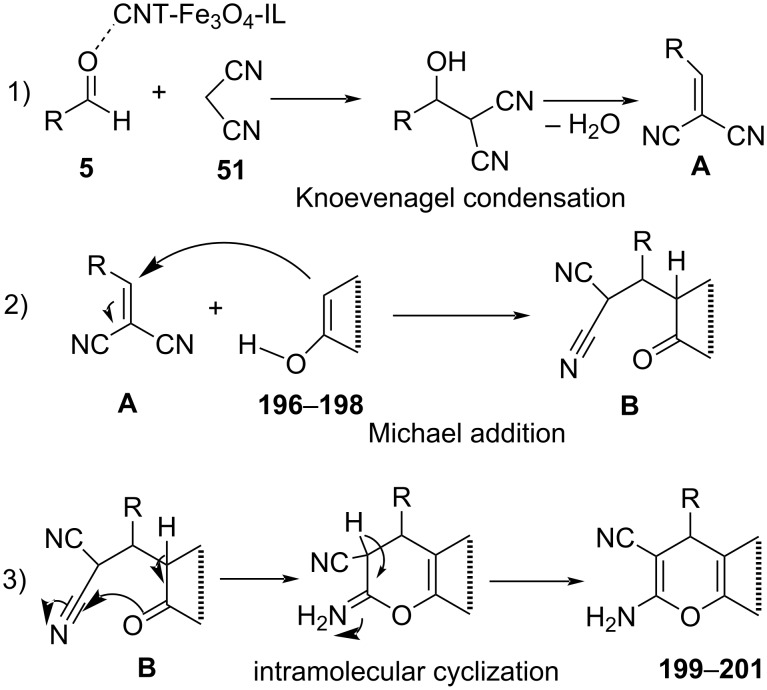
Proposed mechanism for the synthesis of chromenes.

Striving towards chromene-based molecules, Safari and co-worker [160] established a three-component reaction involving substituted phenols **196**–**198**, malononitrile (**51**) and aldehyde **5** using CNT–Fe_3_O_4_–IL as a magnetic nanocatalyst under microwave irradiation in an aqueous medium for the generation of various substituted 2-aminochromenes **199**–**201** (Scheme 81). Ionic liquids can absorb microwave energy and their ability to translate it into homogeneous heat was efficiently demonstrated in this protocol for the generation of the desired molecules. The reusability of the heterocatalyst provides an added advantage to the stated strategy with catalytic activity intact up to five successive runs. The catalyst was recovered using an external magnetic field. A plausible reaction mechanism postulated with an imidazolium cation of CNT-Fe_3_O_4_-IL activating the aldehyde followed by a Knoevenagel condensation with malononitrile gives α-cyanocinnamonitrile derivative **A** by ionic liquid anion. A Michael addition ensued between the activated phenols **196**–**198** and **A** provides **B**. Nucleophilic attack of the phenoxide group on the cyano group led to an intramolecular cyclization of product **B** which finally went through tautomerization to afford the desired products **199**–**201** (Scheme 82).

## Conclusion

In summary, MWA-MCRs provide an easy access to biologically relevant molecules ranging from simple fused rings to complex steroidal molecules. This efficient merger stands as a classical example of technology-driven molecules. The MCR demonstrates an amalgamation of sub-reactions such as Knoevenagel reaction, Michael addition, cycloaddition reaction etc., in a one-pot manner to reassure the atom economy of the reaction for an environmentally benign approach. Whilst MW assistance reduces the time from hours to minutes and even to seconds with higher yields avoiding tedious purification process. The last decade has witnessed an accelerated interest in MWA-MCR to develop molecules and has hastened the process of drug discovery. Continuous efforts can cater towards development of novel approaches in generation of relevant phamacophores with a greener synthetic protocol. This review illustrates various strategies used to generate pharmacologically relevant heterocyclic molecules, such as pyrimidines, pyranes, purines, pyridines, acridine, etc., aided by MW-MCR along with their mechanistic approach. Undoubtedly, there is still an immense possibility for exploration in this field and a lot remains to be brought on the table in the near future. Therefore, this review may pave a direction for many researchers and propel them to investigate and develop newer chemical entities based on MWA-MCRs.
